# Differential Dose- and Tissue-Dependent Effects of foxo on Aging, Metabolic and Proteostatic Pathways

**DOI:** 10.3390/cells10123577

**Published:** 2021-12-18

**Authors:** Maria S. Manola, Sentiljana Gumeni, Ioannis P. Trougakos

**Affiliations:** Department of Cell Biology and Biophysics, Faculty of Biology, National and Kapodistrian University of Athens, Panepistimiopolis, 15784 Athens, Greece; mmanola@biol.uoa.gr (M.S.M.); sgumeni@biol.uoa.gr (S.G.)

**Keywords:** FOXO, *Drosophila*, Nrf2/cncC, proteasome, proteostasis network

## Abstract

Aging is the gradual deterioration of physiological functions that culminates in death. Several studies across a wide range of model organisms have revealed the involvement of FOXO (forkhead box, class O) transcription factors in orchestrating metabolic homeostasis, as well as in regulating longevity. To study possible dose- or tissue-dependent effects of sustained *foxo* overexpression, we utilized two different *Drosophila* transgenic lines expressing high and relatively low *foxo* levels and overexpressed *foxo*, either ubiquitously or in a tissue-specific manner. We found that ubiquitous *foxo* overexpression (OE) accelerated aging, induced the early onset of age-related phenotypes, increased sensitivity to thermal stress, and deregulated metabolic and proteostatic pathways; these phenotypes were more intense in transgenic flies expressing high levels of *foxo*. Interestingly, there is a defined dosage of *foxo* OE in muscles and cardiomyocytes that shifts energy resources into longevity pathways and thus ameliorates not only tissue but also organismal age-related defects. Further, we found that *foxo* OE stimulates in an Nrf2/cncC dependent-manner, counteracting proteostatic pathways, e.g., the ubiquitin-proteasome pathway, which is central in ameliorating the aberrant *foxo* OE-mediated toxicity. These findings highlight the differential dose- and tissue-dependent effects of foxo on aging, metabolic and proteostatic pathways, along with the foxo-Nrf2/cncC functional crosstalk.

## 1. Introduction

Aging is a complex phenomenon caused by the time-dependent loss of cellular homeodynamics and, consequently, physiological organismal functions. The development of age-related phenotypes is a complex stochastic process characterized by the numerous evolutionary conserved hallmarks of aging, which (among others) include loss of energy balance and disruption of proteome homeostasis (or proteostasis) [1,2]. One of the main metabolic pathways involved in aging is the insulin/insulin-like growth factor (IGF-1) signaling (IIS) pathway, whose major function is to coordinate nutrient distribution and utilization throughout the body [3]. Since the initial discovery that mutations in IIS-related genes extend lifespan [4], several research groups have shown that either pharmacological or genetic IIS downregulation extends lifespan in a wide variety of model organisms [5]. Another vital process for organismal homeostasis is maintenance of proteostasis, which has been proven to be central in decelerating the onset of age-related phenotypes; proteostasis is ensured by proteome quality control mechanisms [6] that refer to a highly integrated multilevel system, also known as the proteostasis network (PN) [7]. The central PN regulating components, i.e., the molecular chaperones network, the autophagy-lysosome (ALP), and the ubiquitin-proteasome (UPP) pathways, along with the Nrf2-Keap1 antioxidant responses pathway [8], have been linked to healthspan/lifespan regulation. ALP is mostly involved in the degradation of protein aggregates and damaged organelles, while UPP ensures protein synthesis quality control and degradation of short-lived regulatory or damaged polypeptides [9]. The disruption of the major PN signaling cascades or the deregulation of the PN regulators leads to PN impairment and consequently to increased risk for premature aging [10].

Amongst the pleiotropic stress sensors that have been implicated in the modulation of longevity are the forkhead box-O (FOXOs) transcriptional factors. The FOXO family is a subclass of forkhead transcription factors characterized by a winged-helix DNA-binding domain (DBD), known as a forkhead box. *Caenorhabditis elegans* and *Drosophila melanogaster* possess one FOXO gene (*daf-16* and *foxo*, respectively), whereas, in mammals, there are four members (*FOXO1*, *FOXO3*, *FOXO4*, and *FOXO6*) that regulate gene expression in a tissue-specific pattern [11]. In the presence of insulin and/or IGF-1 signaling, the phosphoinositide 3 kinase (PI3K)/protein kinase B (AKT) signaling cascade is activated, resulting in FOXO phosphorylation and repression of its transcriptional activity [12]. In the absence of insulin and/or IGF-1 signaling or upon cellular (oxidative or metabolic) stress, FOXOs translocate to the nucleus and trigger FOXO-dependent transcriptional programs, which activate damage repair mechanisms, cell fate decisions, stress resistance, and metabolic reprogramming [12,13]. The FOXO-related molecular mechanisms that regulate longevity are not fully understood, but it appears that FOXOs can stimulate longevity-associated gene expression programs [14]; also, they may activate downstream targets by direct interaction or via epigenetic regulation [15].

Reportedly, tissue-specific interventions that alter the expression levels of *foxo* may prolong the health span and/or lifespan of *Drosophila* flies [16,17,18]; however, results can be affected by the drivers’ expression pattern and/or the inducers’ administration procedure [19,20]. *Drosophila* is a tractable model organism characterized by a wide range of tissue types and observable behaviors, the availability of vast genome data and powerful genetic tools, as well as by significant gene homology with disease-related human genes [21,22].

Here we used complementary gain- and loss-of-function approaches in the fly model to provide insights on the possible dose- or tissue-dependent effects of sustained *foxo* overexpression (OE). We found differential dose- and tissue-dependent effects of foxo on aging, metabolic, and proteostatic pathways, along with a foxo-Nrf2/cncC functional crosstalk.

## 2. Materials and Methods

### 2.1. Drosophila Stocks and Maintenance

*Drosophila* flies were maintained at 24 °C, 60% relative humidity on a 12 h light: 12 h dark cycle and fed on a standard medium [23]. Wild-type w^1118^ (+/+) (BDSC 5905), transgenic *foxo*^H^ [UAS *foxo* (BDSC 9575)] and *foxo*^L^ [UAS *foxo* (BDSC 42221)] lines, along with flies expressing the muscle-specific (high transgene expression) Mef2^Gal4^ (BDSC 27390) driver were obtained from the Bloomington *Drosophila* Stock Center (University of Indiana, Bloomington, IN, USA). The muscle-specific (weak expression) driver Mhc^Gal4^ was kindly provided by Dr. Orso (University of Padova, Padova, Italy). The *cncC* knockdown (UAS *cncC*^RNAi^), the *cncC* overexpressing (UAS cncC) line and the Tub^Gal4^ GeneSwitch (tubGSGal4) driver were a gift from Prof. Bohmann (University of Rochester, New York, NY, USA); the conditional driver Tub^Gal4^ is ubiquitously activated upon dietary administration of 320 μΜ RU486 (#M8046, Mifepristone, Merck KGaA, Darmstadt, Germany). Cardiomyocyte-specific tinC.Δ4^Gal4^ driver was donated by Prof. Frasch (Friedrich Alexander University, Erlangen-Nuremberg, Germany).

### 2.2. Flies Sorting, Tissue Microdissections, and Hemolymph Isolation

Flies were sorted under CO_2_ anesthesia 24 h before the experiment, and an equal number of female and male flies were used per assay, unless otherwise stated. As described before, gonads display a distinct vs. somatic tissues aging rate and regulation of major proteostatic components [24]. Hence, isolated somatic tissues (head and thorax) of young, middle-aged, or aged flies (according to the lifespan of each tested group, see below) were analyzed, unless otherwise stated. Thoracic flight muscle, abdomen fat body, heart tissues, intestines, and hemolymph were isolated as previously described [25,26,27].

### 2.3. Gustatory Assay, Body Mass Measurement, and Progeny

Gustatory assay was performed in middle-aged flies (5 female flies) as described before [28], and flies’ abdomens were lensed and captured using a Leica M205 FA stereoscope (Leica Microsystems, Wetzlar, Germany). Flies’ body weight (body mass per fly measured in gr) (10 female flies) and progeny (number of offspring per laid eggs) were assayed as previously described [26].

### 2.4. Locomotion and Longevity

Locomotion (climbing activity) and lifespan (longevity assay) were assayed as described before [26]; at least 40 flies were analyzed in each biological repeat. For survival curves and calculation of median lifespan, we used SPSS software (Statistical Package for Social Sciences-IBM SPSS; version 19.0 for Windows, New York, NY, USA) followed by the Kaplan–Meier and log-rank Mantel–Cox tests for statistical analyses (reported in Table S1).

### 2.5. Stress Sensitivity Assays

#### 2.5.1. Heat Sensitivity Assay

Heat sensitivity assay was performed as described before [26] with minor modifications. In short, groups of 20 flies were placed in plastic vials containing only water immersed Whatman paper to avoid dehydration and incubated at 40 °C for 10 min. Mortality and recovery rates (expressed in purposeful movements) were recorded for the next 90 min at 25 °C using a Leica M205 FA stereoscope (Leica Microsystems, Wetzlar, Germany).

#### 2.5.2. Cold Sensitivity Assay

Cold sensitivity assay was performed as previously described [29] with minor modifications. Briefly, 20 flies were placed in plastic vials containing standard medium and incubated at 4 °C overnight (16 h). The next day, mortality, and recovery rates (expressed in purposeful movements) were captured at 25 °C for 30 min using a Leica M205 FA stereoscope (Leica Microsystems, Wetzlar, Germany).

#### 2.5.3. Nutrients’ Sensitivity Assays

Sensitivity to metabolic stress was firstly assayed by studying flies’ tolerance to starvation. More specifically, young, middle-aged, or aged flies (20 individuals per age group) were transferred in empty vials containing 2 cm of cotton saturated in sterilized water to avoid deaths from dehydration. Vials were maintained under standard housing conditions, and starvation resistance was recorded as the percent of the survived flies, 16 h post starvation. Further studies on metabolic stress tolerance included longevity assays under nutrient deprivation or supplementation. In these assays, 40 flies were cultured in calories-restricted medium, protein-restricted medium, and sugar-over supplemented medium; in all cases longevity assays were performed as described above.

### 2.6. Total RNA Extraction and Quantitative Real-Time (Q-RT-PCR) Analyses

Total RNA was extracted from dissected tissues of 10 flies and converted to cDNA using the FastGene Scriptase II cDNA Kit (#LS53, Nippon Genetics Europe Co. Ltd., Duren, Germany). For Real Time-Q-PCR analysis, HOT FIREPol^®^ EvaGreen^®^ qPCR Mix Plus (#08-36-00001, Solis BioDyne, Tartu, Estonia) was used. Primers were designed using the primer BLAST tool (http://www.ncbi.nlm.nih.gov/tools/primer-blast, accessed on 17 September 2021) and were as reported before [25,26]. The ribosomal gene *RpL32* (also known as *Rp49*) was used as an input reference.

### 2.7. Isolation of Total Protein, Immunoblotting Analyses and Detection of Carbonyl Groups

Isolation of total protein lysates from tissues dissected from 10 flies, immunoblotting analyses, and detection of total carbonylated proteins (#s7150, Millipore, Merck KGaA, Darmstadt, Germany) were performed in duplicate as previously described [25]. Protein expression levels were quantified vs. the respective controls set to 100%. Gapdh was used as a loading control.

### 2.8. Measurement of Reactive Oxygen Species (ROS), 26S Proteasome CT-L/C-L and Cathepsins Activity, as well as of Tissues’ Sugar Content

ROS levels, 26S CT-L and C-L proteasome activity levels, and cathepsins’ activity in total protein lysates isolated from somatic or muscle tissues of 10 flies were measured as described before [30]. Circulating or tissue sugar levels [trehalose (TRE), glucose (GLU), and glycogen (GLY)] were measured as reported before [31]. For all measurements, fluorescence or absorbance was recorded in a Spark^®^ Tecan microplate reader (Tecan Group Ltd., Maennedorf, Switzerland) and expressed as (%) values vs. the respective control groups set to 100%.

### 2.9. CSLM and Immunofluorescence Staining

Dissections, fixation, and immunostaining of tissues were performed as reported before [25,26,27]. At least 10 flies were analyzed per each experimental repeat. Samples were then mounted and viewed using a Digital Eclipse Nikon C1 confocal laser scanning microscope (CLSM) (Nikon Corporation, Tokyo, Japan). Image capture was done using the EZC1 acquisition, and images were analyzed with the CLSM (Nikon Corporation, Tokyo, Japan) or the ImageJ software (Fiji ImageJ software, NIH, New York, NY, USA).

### 2.10. Antibodies

The anti-dFOXO (#CAC-THU-A-DFOXO) antibody was purchased from Cosmo Bio Ltd. (Tokyo, Japan). The antibody against the Prosβ5 proteasome subunit was kindly provided by Maria Figueiredo-Pereira (Hunter College, New York, NY, USA), and the antibody against PSMD11 (Rpn6, #NBP1-46191) was from Novus Biologicals (Centennial, CO, USA). Primary antibodies against 20S-(α) (20Sa, #sc-65755), Rpn10 [26S Proteasome p54 (28), #sc-65748] proteasome subunits and ubiquitin (Ub, #sc-8017) were purchased by Santa Cruz Biotechnology, Inc. (Dallas, TX, USA). Antibodies against _p_Akt1^Ser505^ [phosphorylated *Droso*-Akt (Ser505), #4054S], Akt1 (Akt, #9272S), _p_sgg^ser21/9^ [phosphorylated GSK-3a/b (Ser21/9), #9331S] and GABARAP (#13733S) were from Cell Signaling Technology, Inc. (Danvers, MA, USA). Anti-ATP5a antibody (complex V subunit-ATP5A, #ab14748) was purchased from Abcam (Cambridge, UK); anti-sgg antibody (Anti-GSK3, #05-412) was from Millipore (Merck KGaA, Darmstadt, Germany), the anti-InR antibody was kindly provided by Prof. Ernst Hafen (ETH, Zurich, Switzerland) and the antibody against Gapdh (#G9545) was from Sigma-Aldrich (Merck KGaA, Darmstadt, Germany). Secondary antibodies, i.e., Alexa Fluor^®^ 647 AffiniPure Donkey Anti-Rabbit IgG (#711-605-152) and Alexa Fluor^®^ 647 AffiniPure Donkey Anti-Mouse IgG (#715-605-151) were from Jackson ImmunoResearch Europe Ltd. (Ely, UK). Fluorescent dyes, Bodipy (#D3922), Phalloidin (#R415), and DAPI (#D1306) were purchased by Molecular Probes™ (Thermo Fisher Scientific Inc., Waltham, MA, USA).

### 2.11. Statistical Analyses

The presented experiments were analyzed at least in triplicates unless otherwise indicated. Data points correspond to the means of the independent experiments; error bars denote standard deviation (SD), and differences between compared groups were evaluated using the independent (unpaired) *t-*test analysis or a two-way ANOVA test followed by Tukey’s multiple comparisons test. For graphical representation of data and statistical analyses, MS Excel (Microsoft Office, Washington, DC, USA), the Statistical Package for Social Sciences (IBM SPSS, version 23.0 for Windows, New York, NY, USA), and GraphPad Prism 8.00 (GraphPad Software, San Diego, CA, USA) were used. Significance was accepted at * p < 0.05 (shown in graphs by one asterisk); two asterisks denote ** p < 0.01.

## 3. Results

### 3.1. Prolonged Ubiquitous foxo OE Accelerates Aging Phenotypes and Impairs Thermal Stress Responses Dose-Dependently

To better understand the functional implication of *foxo* in longevity and proteostasis, we sought to investigate the effects of inducible ubiquitous (Tub^Gal4^) *foxo* OE in *Drosophila* flies. We used two *Drosophila* lines, i.e., stock N^o^ 9575 (hereafter referred to as *foxo*^H^) and stock N^o^ 44,214 (hereafter referred to as *foxo*^L^) (Figure S1a), which express different *foxo* levels vs. controls (i.e., w^1118^ or non-induced flies). Specifically, *foxo*^H^ transgenic flies express significantly higher *foxo* mRNA and foxo protein levels as compared to *foxo*^L^ flies (Figure S1b–d), and thus these lines can be used to study likely FOXO gene dosage-dependent effects on flies’ physiology.

**Figure 1:**
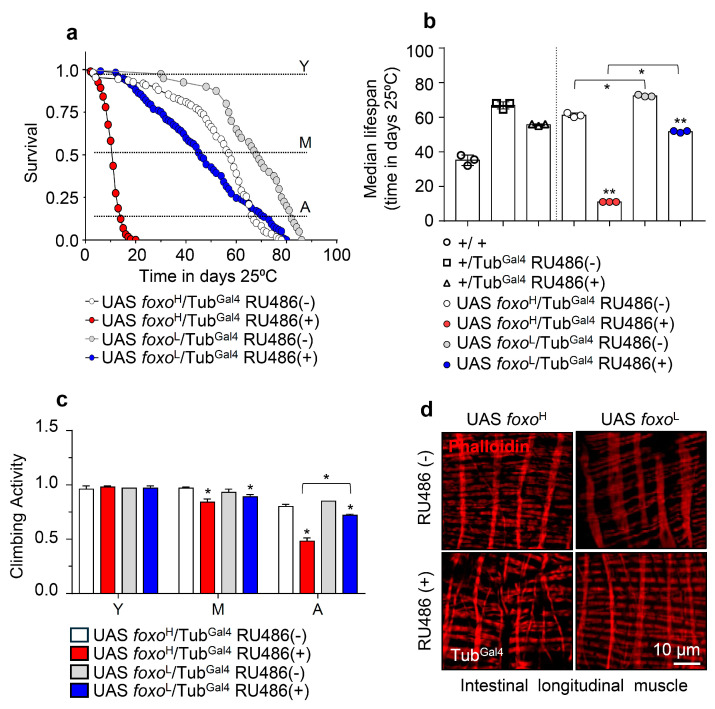
Ubiquitous *foxo* OE accelerates aging phenotypes in a dose-dependent manner. (**a**) Survival curves of *foxo*^H^ and *foxo*^L^ overexpressing flies and (**b**) median lifespan of *foxo* overexpressing flies vs. respective controls [i.e., +/+ (w^1118^ flies), +/Tub^Gal4^ RU486(−) and *+*/Tub^Gal4^ RU486(+) flies]. Statistics of longevity assays are shown in Table S1. (**c**) Locomotion (climbing activity) of young (Y), middle-aged (M), and aged (A) *foxo* overexpressing flies vs. controls. (**d**) Representative CLSM images of intestinal longitudinal muscle fibers’ actin filaments (Phalloidin stain) of female middle-aged *foxo* overexpressing flies vs. control. Bars, ±SD; n ≥ 3, ≥10 flies were analyzed per experimental repeat. Statistical significance was measured with unpaired *t*-test, * *p* < 0.05, ** *p* < 0.01.

Given FOXO’s implication in longevity [16,17,18,19], we initially analyzed the survival rates of the *foxo*^H^ and *foxo*^L^ transgenic lines after inducible ubiquitous (Tub^Gal4^) *foxo* OE. Longevity assays revealed that *foxo* OE accelerated aging dose-dependently since *foxo*^H^ flies had a ~80% reduction (vs. control) of their median lifespan, whereas *foxo*^L^ showed a ~40% reduction in their median lifespan (Figure 1a,b; see also Table S1). Notably, *foxo* OE at very low levels, e.g., because of leaky transgene expression in non-induced *foxo*^L^ flies, increased flies’ longevity, and median lifespan vs. control (Figure 1b), further highlighting the impact of dose (and likely duration) on *foxo* OE-mediated effects.

Moreover, we found that both the *foxo*^H^ and *foxo*^L^ transgenic lines exhibited a significant acceleration of the age-related decline in neuromuscular activity, which was more intense (and likely earlier) in *foxo*^H^ flies (Figure 1c). In support, visualization of the longitudinal muscle fibers’ integrity isolated from middle-aged flies’ midguts revealed severe muscle fiber breakages (a marker of premature aging) in induced *foxo*^H^ flies, while midgut muscle fibers were mildly impaired in *foxo*^L^ flies (Figure 1d and Figure S2a).

To further investigate the effects of *foxo* OE on flies’ physiology, we also assayed transgenic flies’ responses to thermal stress. We found that *foxo* OE increased (vs. non-induced flies) susceptibility (mortality and recovery rate after treatment) of flies to both cold and heat (a condition of, among others, increased proteome instability) stress (Figure S3); again, the *foxo*^H^ line was more sensitive to either cold or heat stress.

Taken together, ubiquitous foxo overactivation induces (dose-dependently) the early onset of aging phenotypes and saturates the buffering capacity of stress response pathways.

### 3.2. Sustained foxo OE Perturbs Cellular Energetics and Reduces Tolerance to Nutritional Deprivation

**Figure 2:**
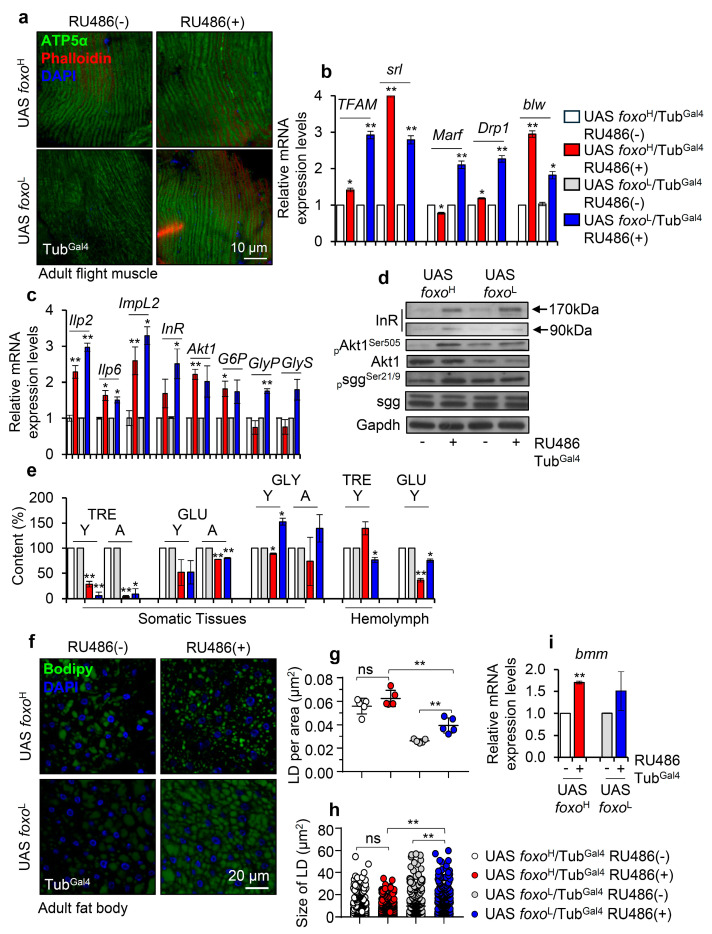
Perturbation of major bioenergetic and metabolic signaling pathways after ubiquitous *foxo* OE in *Drosophila* flies. (**a**) Representative CLMS images of female adult middle-aged *foxo* overexpressing, or control flies’ muscle fibers stained with anti-ATP5a (mitochondrial subunit). (**b**) Relative expression levels of mitochondrial (*TFAM*, *srl/PGC1α*, *Marf*, *Drp1*, *blw/ATP5a*) genes in shown transgenic flies. (**c**) Relative expression levels of *Ilp2*, *Ilp6*, *ImpL2*, *InR*, *Akt1*, *G6P*, *GlyP*, and *GlyS* genes (bars are as in **b**) and, (**d**) representative immunoblotting analysis showing expression levels of insulin receptor (InR), _p_Akt1^Ser505^ (phosphorylated activated form), total Akt1, _p_sgg^Ser21/9^ (phosphorylated inhibitory form) and total sgg in indicated *foxo* overexpressing flies; Gapdh probing was used as a loading reference. (**e**) Content (%) of TRE (trehalose), GLU (glucose), and GLY (glycogen) in dissected somatic tissues or isolated hemolymph from middle-aged *foxo* overexpressing transgenic flies (bars are as in **b**); controls were set to 100%. (**f**) Representative CLSM images of lipid droplets (LDs) (Bodipy staining, green); nuclei were stained with DAPI (blue); (**g**) quantification of LDs’ number [LDs per area (μm^2^)] and, (**h**) measurement (in μm^2^) of LDs’ size in the fat bodies of female middle-aged *foxo* overexpressing transgenic flies. (**i**) Relative mRNA expression levels of *bmm* (also known as *ATGL*) after *foxo* OE. In (**b**–**d**,**i**), expression of transgenes was induced by 320 μΜ RU486 for 7 days; data refer to dissected young flies’ somatic tissues. In (**b**,**c**,**i**), gene expression was plotted vs. controls set to 1; the *RpL32* gene expression was used as input reference. Bars, ±SD; n ≥ 2, 10 flies were analyzed per experimental repeat; significant differences were calculated with unpaired *t*-test (**b**,**c**,**e**,**i**) or two-way ANOVA followed by Tukey’s multiple comparisons test (**g**,**h**), * *p* < 0.05, ** *p* < 0.01.

Since foxo has also been functionally implicated in energy regulation and metabolism [12,13], we then studied the impact of *foxo* OE on mitostatic and bioenergetic pathways. *foxo* expression levels did not significantly affect mitochondria structure in the flight muscle of young transgenic flies (Figure 2a); while both the *foxo*^H^ and *foxo*^L^ lines had upregulated expression of mitochondrial biogenesis (*TFAM*, *srl*), dynamics (*Marf*, *Drp1*), and energetics (*blw*) genes (Figure 2b). Interestingly, *foxo* OE also likely deregulated major IIS and glucose metabolism modules, as we noted in both *foxo* overexpressing lines the induction of the insulin-like peptides (*llp2*, *Ilp6*), Ilp2 inhibitory ecdysone (*ImpL2*), insulin receptor (*InR*), *Akt1*, and the gluconeogenesis related (glucose-6-phosphate, *G6P*) genes; notably, the glycogen phosphorylase (*GlyP*) and glycogen synthase (*GlyS*) genes are only upregulated in the *foxo*^L^ transgenic line (Figure 2c). Consistently, immunoblotting analysis of tissues’ lysates probed with antibodies against insulin receptor (InR), activated (phosphorylated Akt1^Ser505^) and total Akt1; the inhibitory form of sgg (phosphorylated sgg^Ser21/9^) and total sgg, revealed the activation of the IIS pathway in *foxo*^H^ flies and a trend towards activation in *foxo*^L^ flies (Figure 2d and Figure S2b). Thus, *foxo* OE triggers (among others) a counteractive response which via activation of the IIS pathway aims to suppress its transcriptional activity [12].

Next, we investigated the content of sugars in isolated somatic tissues and hemolymph of both young and aged control and transgenic flies. We found that trehalose (TRE), a principal circulating sugar in hemolymph that serves as an efficient flight fuel due to its higher vs. glucose (GLU) [32] energy yield, was decreased in the somatic tissues of both transgenic lines and in the hemolymph of *foxo*^L^ flies (Figure 2e). Also, *foxo* OE suppressed (in both lines) GLU levels in somatic tissues and hemolymph, while glycogen (GLY) levels were found to decrease (likely due to increased breakdown) in *foxo*^H^ and increase in *foxo*^L^ transgenic flies (Figure 2e). Moreover, staining of an alternative source for energy storage, i.e., lipid droplets (LDs) in flies’ fat body tissues (Figure 2f), showed that the LDs in *foxo* overexpressing flies tended to increase in number but to be smaller in size as compared to control; these readouts were more intense in *foxo*^L^ flies since even non-induced *foxo*^H^ flies already showed intense lipolysis probably due to leaky transgene expression, once again underlying the importance of gene-dosage in foxo-induced phenotypes (Figure 2g,h). Consistently, *foxo* OE in both lines triggered a dose-dependent upregulation of *bmm* (also known as *ATGL*) (Figure 2i), which enhances fat storage depletion under nutritional deprivation [33].

**Figure 3:**
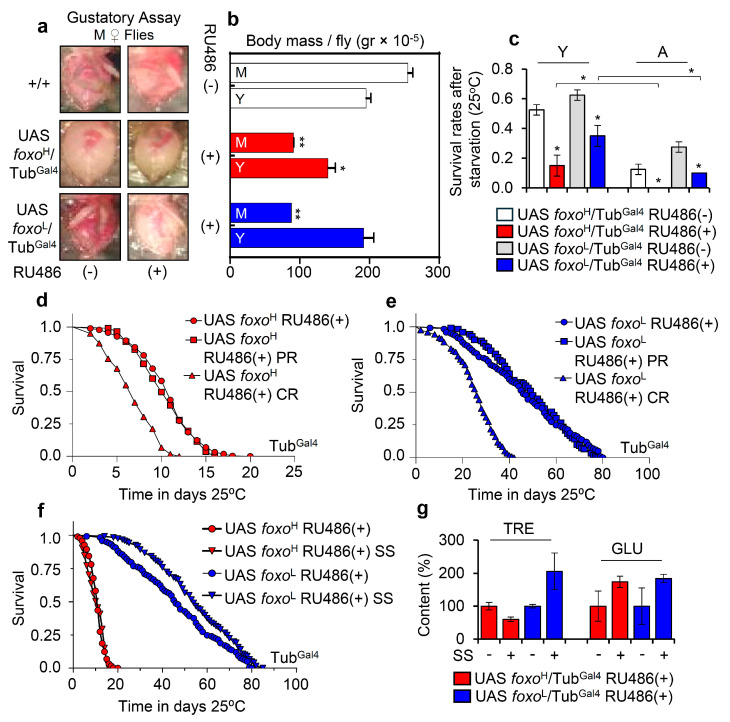
*foxo* OE exhausts flies’ energy stores, reducing tolerance to nutritional deprivation; a phenotype that can be partially rescued by increased calories intake. (**a**) Representative dissected abdomens from middle-aged (M) female *foxo* overexpressing flies vs. controls (including w^1118^ flies) following gustatory assay to test food intake. (**b**) Measurement of body mass per fly (gr × 10^−5^) of young (Y) and middle-aged (M) *foxo* overexpressing flies (bars are as in **c**). (**c**) Survival rates after starvation of young (Y) or aged (A) *foxo* overexpressing transgenic flies. (**d**,**e**) Longevity curves of shown *foxo* overexpressing transgenic flies after nutritional deprivation (CR, calories restriction; PR, protein restriction). (**f**) Longevity curves of *foxo* overexpressing flies following sugar over-supplementation (SS). Statistics of longevity assays are reported in Table S1. (**g**) Relative content of TRE and GLU in isolated somatic tissues of aged *foxo* overexpressing flies after sugar over-supplementation; control values were set to 100%. Bars, ± SD; n ≥ 3, ≥ 5 flies were analyzed per experimental repeat. Statistical significance in (**b**,**c**,**g**) was calculated with unpaired *t*-test, * *p* < 0.05, ** *p* < 0.01.

Taking into consideration the hypoglycemic and lipolytic state of *foxo* overexpressing flies’ tissues and the fact that foxo is an important regulator of IIS in the peripheral tissues and in regulating food intake [34], we then analyzed the food consumption and body weight of *foxo* overexpressing flies. We found that *foxo* OE reduces feeding rates (Figure 3a) and body weight (body mass/fly) (Figure 3b) in a dose-dependent manner. Further, we tested the tolerance of *foxo* overexpressing flies to metabolic stress by firstly exposing them to starvation. We found that *foxo* OE increased (dose-dependently) flies’ sensitivity to nutrients deprivation (Figure 3c). Moreover, we observed that while calories restriction (CR) was toxic for both *foxo* transgenic lines, protein restriction (PR) did not affect significantly their median lifespan (Figure 3d,e). Given that *foxo* overexpressing flies had reduced somatic and circulating sugar levels (see above), we then supplemented flies’ culture medium with additional sugar. Interestingly, sugar over-supplementation (SS) significantly increased (vs. control) by ~20% the median lifespan of *foxo*^L^, but not of *foxo*^H^, line (Figure 3f). Since the increase in *foxo*^L^ flies’ median lifespan following additional sugar supplementation was paralleled by increased TRE and GLU tissue levels (Figure 3g), it is likely that the hypoglycemia induced after ubiquitous *foxo* OE is a key factor to reduced longevity.

These findings indicate that ubiquitous *foxo* OE progressively and dose-dependently deregulates the main components of mitostatic and bioenergetic signaling cascades; interestingly, foxo also likely controls appetite and cellular responses to nutritional deprivation.

### 3.3. While High Levels of Muscle- or Cardiomyocytes-Targeted foxo OE Are Toxic, Moderate OE Increases Healthspan and Delays Age-Related Phenotypes

To study whether tissue-specific *foxo* OE can differentially affect flies’ physiology and longevity, we overexpressed *foxo* in muscles by using the Mhc^Gal4^ and Mef2^Gal4^ drivers (which induce moderate and strong transgene expression, respectively), as well as in cardiomyocytes by employing the tinC.Δ4^Gal4^ driver (induces strong transgene expression).

**Figure 4:**
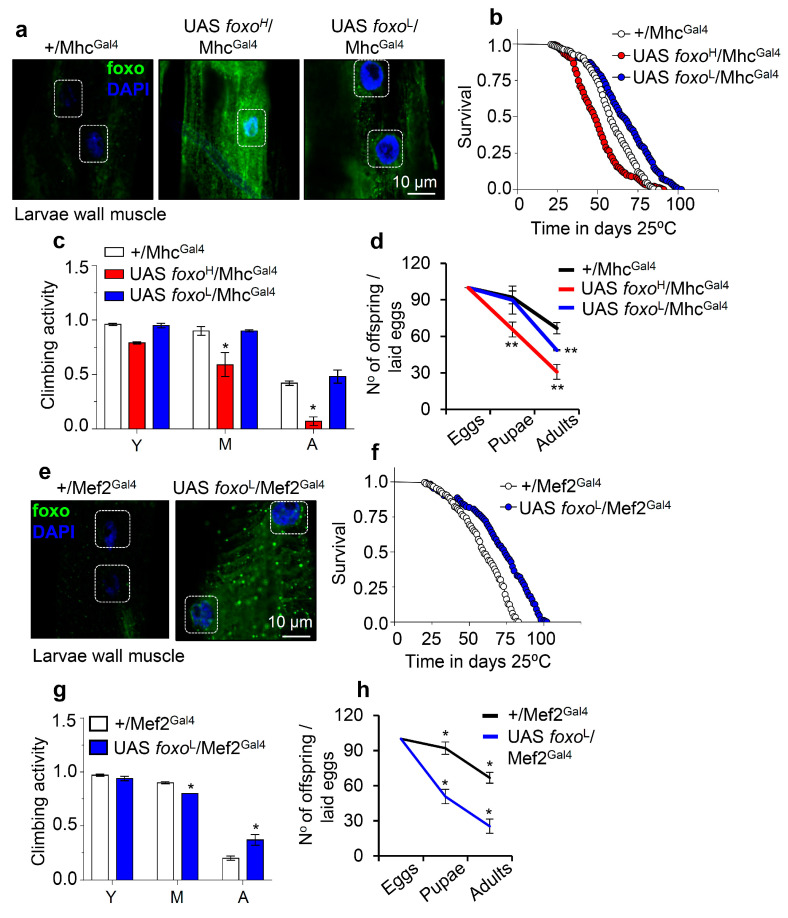
Muscle-targeted *foxo* OE delays aging dose-dependently. (**a**) Representative CLSM images of third instar larvae body wall’s muscle fibers after targeted *foxo* OE in muscles (Mhc^Gal4^); samples were stained with an anti-FOXO antibody and counterstained with DAPI. (**b**–**d**) Longevity (**b**), locomotion performance (climbing activity) of young (Y), middle-aged (M), and aged (A) flies (**c**), and offspring number per laid eggs (**d**), of *Drosophila* flies with the shown genotypes vs. respective controls. (**e**) Representative CLSM images of third instar larvae body wall’s muscle fibers after muscle targeted (Mef2^Gal4^; driver with strong expression) OE of *foxo*^L^; samples were stained with an anti-FOXO antibody and counterstained with DAPI. (**f**–**h**) Lifespan (**f**), locomotion performance (climbing activity) at indicated ages (**g**), and offspring number per laid eggs (**h**) of the shown transgenic flies. Statistics of longevity assays are reported in Table S1. Bars, ± SD; n ≥ 3, ≥ 10 flies were analyzed per experimental repeat. In (**c**,**d**,**g**,**h**) significant differences were calculated with the unpaired *t*-test, * *p* < 0.05, ** *p* < 0.01.

Staining of larvae expressing *foxo*^H^/Mhc^Gal4^ revealed both nuclear and cytoplasmic foxo localization in muscle fibers (Figure 4a and Figure S4), while adult flies expressing *foxo*^H^/Mhc^Gal4^ had reduced (as compared to control) longevity (Figure 4b), accelerated neuromuscular defects (Figure 4c), and reduced adult flies hatching rates (Figure 4d). Notably, *foxo*^L^/Mhc^Gal4^ flies had increased (vs. control) longevity (by ~13%; Figure 4b, Table S1) with no effect on neuromuscular (climbing activity) functionality (Figure 4c); these flies had, however, significantly lower fertility rates vs. control (Figure 4d), indicating that longevity is likely partially promoted at the cost of suppressed reproduction rates.

On the contrary, muscle-specific expression of *foxo*^H^ by using the Mef2^Gal4^ driver was lethal at larval stages (not shown), while *foxo*^L^/Mhc^Gal4^ larvae completed development (Figure 4e and Figure S4), and adults showed increased (vs. control) longevity and median lifespan by ~20% (Figure 4f); in support *foxo*^L^/Mhc^Gal4^ transgenic flies showed a delay in the appearance of age-related impairment of locomotion (Figure 4g). Once again, these physiological adjustments in *foxo*^L^/Mhc^Gal4^ flies decreased fertility rates (Figure 4h). Overall, these findings further support the significance of *foxo* gene expression dosage in regulating all distinct phases of flies’ life cycle.

**Figure 5:**
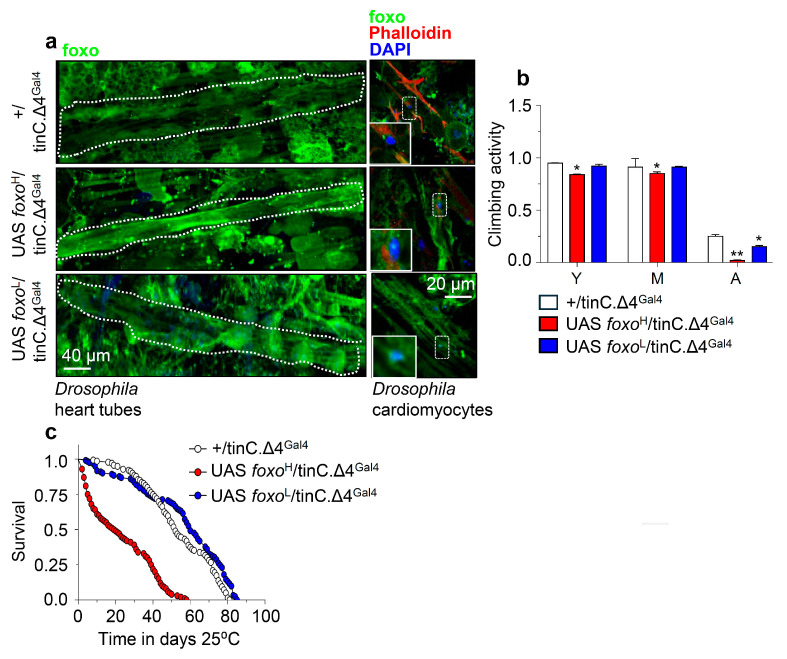
Cardiomyocytes-targeted *foxo* OE induces heart-tissue remodeling and impacts flies’ physiology. (**a**) Representative CLSM images of intact young *Drosophila* heart tubes and of cardiomyocytes following *foxo^H^* or *foxo^L^* heart-specific (tinC.Δ4^Gal4^) OE; samples were stained with an anti-FOXO antibody and counterstained with DAPI to visualize nuclei. (**b**) Locomotion (climbing activity) of young (Y), middle-aged (M), and aged (A) flies of the indicated genotypes and (**c**) longevity of shown transgenic flies after heart-targeted *foxo* OE vs. controls. Statistics of longevity assays are reported in Table S1. Bars, ± SD; n ≥3, ≥ 10 flies were analyzed per experimental repeat. In (**b**) statistical significance was measured with the unpaired *t*-test, * *p* < 0.05, ** *p* < 0.01.

Next, we overexpressed the *foxo*^H^ and *foxo*^L^ transgenes in *Drosophila* cardiac tissue (cardiomyocytes), where foxo is thought to play a crucial role in maintaining proteostasis [35]. Confocal visualization showed that foxo was highly expressed in the heart of *foxo*^H^/tinC.Δ4^Gal4^ flies (Figure 5a and Figure S5); these transgenic flies displayed thinner heart tubes and impaired heart muscle tissue integrity vs. either +/tinC.Δ4^Gal4^ or *foxo*^L^/tinC.Δ4^Gal4^ flies which had a rather physiological cardiac tissue (Figure 5a and Figure S5). Moreover, while *foxo*^H^ OE in cardiac tissues exaggerated neuromuscular defects (Figure 5b) and remarkably shortened flies’ longevity (Figure 5c), indicating heart dysfunction-mediated systemic effects; heart-specific OE of *foxo*^L^ only mildly impaired neuromuscular functionality of aged flies (Figure 5b) and slightly improved median flies’ lifespan (Figure 5c; see also Table S1).

These data further support the dose- and tissue-dependency of *foxo* expression in maintaining tissue integrity and functionality that, in turn, supports systemic homeostasis and delays aging.

### 3.4. Inducible foxo OE Activates Proteostatic Pathways

**Figure 6:**
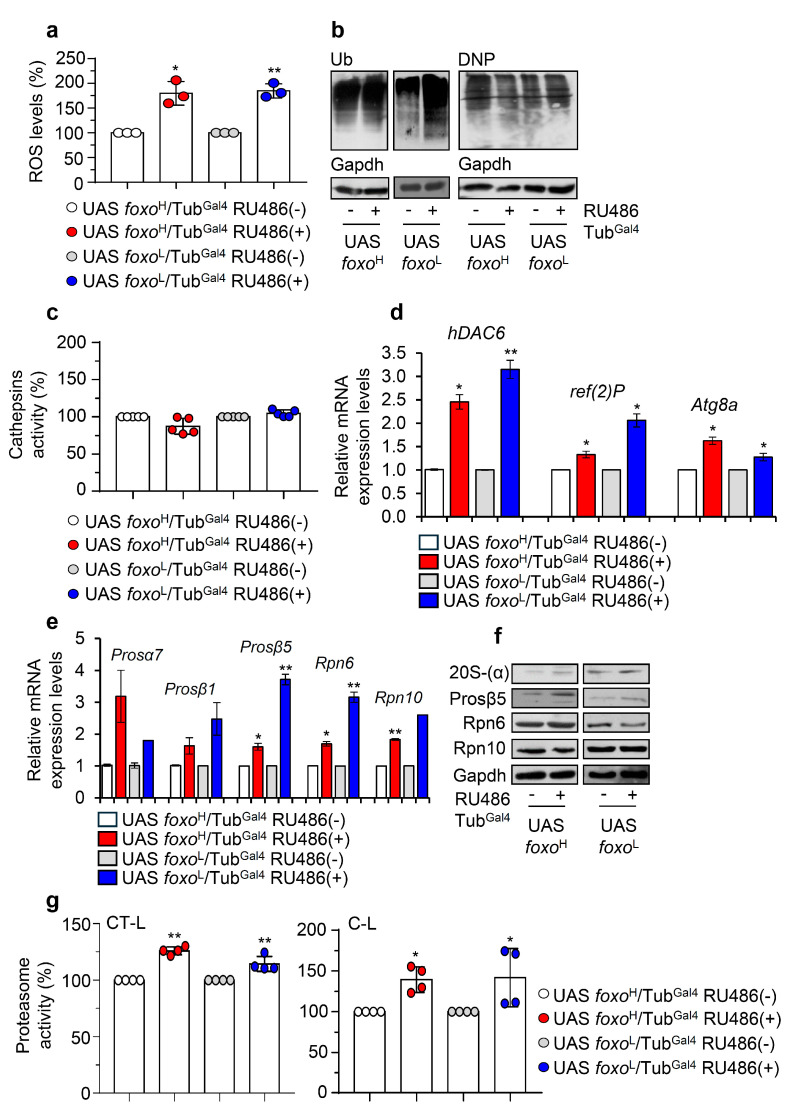
Ubiquitous *foxo* OE causes oxidative and proteome instability triggering proteasome activation. (**a**) ROS (%) levels in *foxo* overexpressing flies vs. controls. (**b**) Representative immunoblots of cellular ubiquitinated (Ub) and carbonylated (DNP) proteins in *foxo* overexpressing flies’ somatic tissues. (**c**) Cathepsins activity (%) in somatic tissues of *foxo* overexpressing flies. (**d**) Relative expression levels of autophagy-related (*hDAC6*, *ref(2)P*, *Atg8a*) and (**e**) proteasomal (*Prosα7*, *Prosβ1*, *Prosβ5*, *Rpn6*, *Rpn10*) genes in shown *foxo* overexpressing transgenic flies’ somatic tissues. (**f**) Immunoblotting analyses showing expression of the 20S-(α), Prosβ5, Rpn6, and Rpn10 proteasome subunits after inducible *foxo* OE in flies’ somatic tissues. (**g**) 26S CT-L and C-L proteasome activities (%) in tissues lysates of shown *foxo* overexpressing transgenic flies’ somatic tissues; transgenes were induced for 7 days in young *Drosophila* flies. In (**a**,**c**,**g**) control values were set to 100%. In (**b**,**f**), Gapdh was used as a loading control. In (**d**,**e**), gene expression was plotted vs. controls set to 1; the *RpL32* gene expression was used as input reference. Bars, ± SD; n ≥ 2, 10 flies were used per experimental repeat; significant differences were calculated with unpaired *t*-test (**d**,**e**) or two-way ANOVA followed by Tukey’s multiple comparisons test (**a**,**c**,**g**), * *p* < 0.05, ** *p* < 0.01.

Considering the suggested regulatory role of foxo on proteostasis [11], we then investigated the impact of *foxo* OE on proteostatic pathways. Ubiquitous *foxo* OE resulted in increased ROS levels (Figure 6a) and accumulating ubiquitinated and carbonylated polypeptides (Figure 6b), suggesting extensive oxidative and proteostatic instability. In support, although we found no significant alternations in cellular cathepsins activity status in either *foxo* overexpressing lines (Figure 6c), we noted in both lines upregulation of antioxidant (not shown) and autophagy-related (*hDAC6*, *ref(2)P*, *Atg8a*) genes (Figure 6d); also, we found a significant induction of proteasomal genes (*Prosα7*, *Prosβ1*, *Prosβ5*, *Rpn6*, *Rpn10*) (Figure 6e) and of most protein subunits [20S-(a), Prosβ5 and Rpn6] (Figure 6f and Figure S6a), as well as of both of chymotrypsin- (CT-L) and caspase- (C-L) like 26S proteasome activities after ubiquitous *foxo* OE (Figure 6g).

Moderate muscle-specific *foxo* upregulation showed no effect on cathepsins or proteasome activities (Figure S6b,c), and despite some accumulation of ubiquitinated peptides, we found no induction of proteome carbonylation (Figure S6d). On the contrary, *foxo*^L^/Mef2^Gal4^ flies had upregulated levels of both cathepsins and proteasome activity (Figure S6e,f); consistently, whole body analyses revealed a significant downregulation of ubiquitinated proteins (Figure S6g). Additional analyses of somatic tissues after targeted *foxo* OE in cardiomyocytes showed a decrease of proteasome activity in *foxo*^H^/tinC.Δ4^Gal4^ flies and an overall increase in *foxo*^L^/tinC.Δ4^Gal4^ flies (Figure S6h).

Overall, our data highlight that either ubiquitous or tissue-specific *foxo* upregulation induces proteostatic modules.

### 3.5. Proteasome Activation in foxo OE Transgenic Flies Is Nrf2/cncC-Mediated

**Figure 7:**
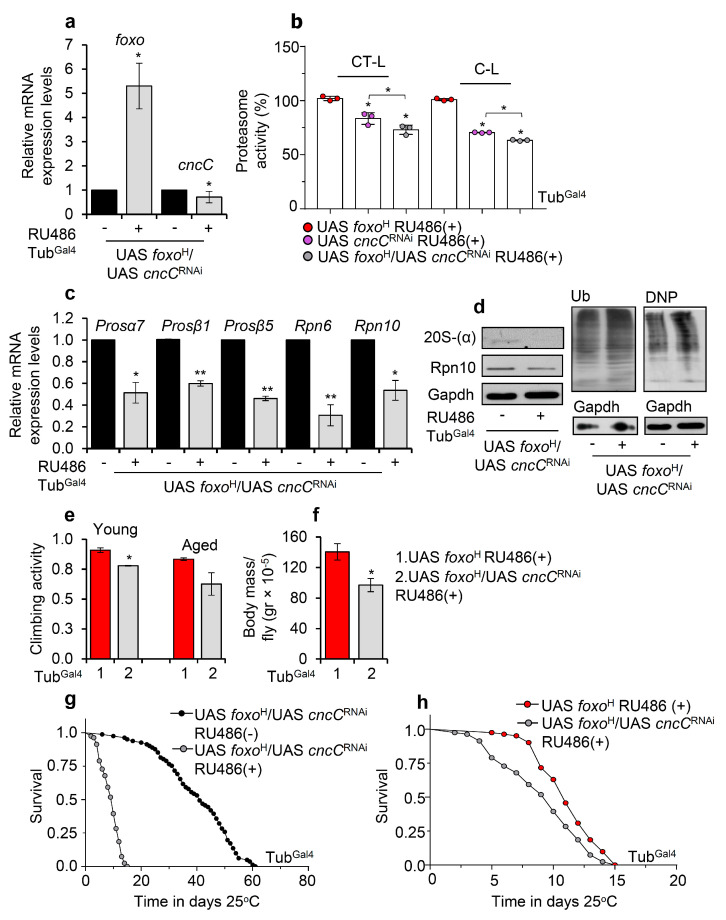
Proteasome activation after *foxo* OE is mediated by Nrf2/cncC. (**a**) Relative expression levels of *foxo* and *Nrf2/cncC* genes in the shown transgenic line after ubiquitous *foxo*^H^/*cncC*^RNAi^ expression. (**b**) 26S (%) CT-L and C-L proteasome activity levels in flies’ somatic tissues after inducible ubiquitous *foxo*^H^/*cncC*^RNAi^ expression vs. the respective control group; control values were set to 100%. (**c**) Relative expression levels (vs. controls) of proteasomal (*Prosα7*, *Prosβ1*, *Prosβ5*, *Rpn6*, *Rpn10*) genes in *foxo*^H^/*cncC*^RNAi^ overexpressing transgenic flies. (**d**) Immunoblotting analyses showing 20S-(α) and Rpn10 (proteasomal subunits) expression levels, as well as total ubiquitinated (Ub) and carbonylated (DNP) proteins in tissue lysates of *foxo*^H^/*cncC*^RNAi^ overexpressing flies; Gapdh was used as a loading control. (**e**) Neuromuscular degeneration (climbing activity) during aging and (**f**) body mass per fly (gr × 10^−5^) of *foxo*^H^/*cncC*^RNAi^ vs. *foxo*^H^ overexpressing flies. (**g**) Longevity curves of *foxo*^H^/*cncC*^RNAi^ overexpressing flies vs. respective controls or (**h**) as compared to *foxo*^H^ overexpressing flies. Statistics of longevity assays are reported in Table S1. In (**a**–**d**,**f**), experiments were performed in young flies 7 days after induction of the transgenes. In (**a**,**c**), gene expression was plotted vs. controls set to 1. The *RpL32* gene expression was used as input reference. Bars, ±SD; n ≥ 2, ≥10 flies were used per experimental repeat. Statistical significance was measured with unpaired *t*-test (**a**,**c**,**e**,**f**) or two-way ANOVA followed by Tukey’s multiple comparisons test (**b**), * *p* < 0.05, ** *p* < 0.01.

Given that the foxo-mediated effects on UPP are dose-dependent, we hypothesized that these are induced by a foxo transcriptional target; in particular, foxo is thought not to act alone, but rather is engaged in an integrated regulatory network and interacts with other transcription factors [36] to mediate its function. As Nrf2 is reportedly one of the main UPP regulators [25], we knocked down ubiquitously *Nrf2/cncC* (*cncC*^RNAi^) in *foxo*^H^ overexpressing flies (Figure 7a) and found a significant decrease (vs. control) of 26S proteasome activities (Figure 7b). Moreover, *cncC*^RNAi^ suppressed the expression of proteasome genes (Figure 7c) and protein subunits (Figure 7d) and increased proteome ubiquitination and carbonylation levels (Figure 7d), suggesting that Nrf2/cncC likely mediates the *foxo* OE induced UPP activation. Moreover, *foxo*^H^/*cncC*^RNAi^ transgenic flies had decreased locomotion activity during aging (Figure 7e) and reduced body weight (body mass/fly) (Figure 7f) as compared to *foxo*^H^ flies; these physiological alternations were followed by an ~80% (Figure 7g) and a ~10% (Figure 7h) decrease in *foxo*^H^*/cncC*^RNAi^ flies median lifespan vs. *foxo*^H^*/cncC*^RNAi^ non-induced and *foxo*^H^ flies, respectively.

In support, concomitant pharmacological inhibition of proteasome in *foxo*^H^ overexpressing flies reduced lifespan, induced the accumulation of ubiquitinated proteins, and triggered a counteracting upregulation of proteostatic modules (Figure S7a,b). Proteasome inhibition in *foxo*^L^ transgenic flies significantly reduced longevity in the absence of significant deregulation of proteostatic modules expression (Figure S7a,b), indicating the pleiotropic dose-dependent effects of *foxo* OE. *Nrf2/cncC* knockdown in *foxo*^H^ transgenic flies treated with the proteasome inhibitor suppressed the upregulation of proteasome inhibition-mediated proteasomal genes upregulation (Figure S7c), further supporting the notion that Nrf2/cncC mediates UPP induction upon aberrant *foxo* activation.

To further validate these findings, we then overexpressed both *Nrf2/cncC* (*cnc*C) and *foxo*^H^ in flies (Figure S8a) and found that combined ubiquitous *foxo*^H^*/cncC* OE resulted in more intense (vs. sole *Nrf2/cncC* or *foxo*^H^ OE) 26S proteasome activities induction (Figure S8b). Moreover, flies co-expressing *Nrf2/cncC* and *foxo*^H^ further upregulated proteasome genes (Figure S8c) and proteasome subunits along with decreased ubiquitination levels, as compared to non-induced flies (Figure S8d). Nonetheless, *foxo*^H^/*cncC* OE reduced locomotion and flies’ weight as compared to sole *foxo*^H^ OE indicating an early induction of aging phenotypes (Figure S8e,f), and similarly to *foxo*^H^*/cncC*^RNAi^ transgenic flies, *foxo*^H^*/cncC* overexpressing flies showed a significant lifespan reduction (Figure S8g,h).

These data, apart from highlighting the functional crosstalk of the Nrf/cncC and foxo transcription factors (stress sensors), also further support the notion of the detrimental effects of stress-responsive elements’ prolonged activation on organisms’ physiology and longevity.

## 4. Discussion

Biological aging results from the progressive accumulation of cellular damage and the diminishing buffering capacity of stress response mechanisms [1]. Genetic or pharmacological interventions that target the stress sensing transcription factor FOXO have long been associated with tissue homeostasis and longevity in several model organisms [37,38]. External stress stimuli trigger a large spectrum of post-translational modifications that stimulate FOXO’s translocation from the cytoplasm to the nucleus, where it acts mostly as a transcriptional activator to regulate metabolic, oxidative, and proteostatic equilibrium [39]. Specificity of FOXO-mediated gene signatures is mainly attributed to a large variety of upstream regulatory elements, a wide range of co-acting transcription factors and gene regulatory complexes, isoform-specific functional divergence, enhancers’ landscape, and genomic 3D architecture of each tissue and cell type [40]. Nevertheless, several aspects of the tissue-dependent FOXO-mediated stress responses cascade, the synergistic action of FOXO with other transcription modulators, and the selection of potential binding sites in the genome remain elusive [3,37,41]. Apart from FOXO predominant transcriptional activity, there have been few recent reports that underlie its non-transcriptional activity [40]. The less well-known non-transcriptional FOXO function(s) relate to the interaction of cytoplasmic FOXO with other sub-cellular modules, such as RPA1 (Replication Protein A1) and ATM, regulating DNA synthesis and DNA damage responses, respectively; or with TSC2 controlling the insulin signaling pathway through mTOR and ATG7 managing the rate of protein degradation through autophagy [42,43,44,45,46]. However, since FOXO also transcriptionally regulates several of the aforementioned cellular processes, it remains to be determined how relevant these non-transcriptional functions are compared to the transcriptional regulation; if they are related to isoform-specific functions, and whether they could be associated with FOXO’s gene dosage since FOXO protein levels impact on the transcription factor’s cellular location, thereby affecting upstream signaling, triggering, for example, complex feedback signaling cascades aiming to suppress FOXO overactivation [40].

Herein we report, that the *foxo* induced longevity strongly relies on its expression dynamics. We found that ubiquitous *foxo* OE accelerated aging, induced the early onset of age-related phenotypes, and increased sensitivity to thermal stress in a dose-dependent manner. Similarly, ubiquitous high expression levels of other transcription factors functioning as stress sensors, e.g., Nrf2/cncC, have detrimental effects on flies’ longevity [25]. Several studies support the notion that the exhaustion of stress sensing mechanisms after chronic robust activation diminishes lifespan by shifting resources from survival towards somatic preservation which, in turn, may favor the early onset of age-related disorders [3,20,25].

Sustained foxo activation deregulated nutrient and energy-sensing mechanisms in a dose-dependent manner, increased sensitivity to nutrient deprivation, and phenocopied acute starvation since it exhausted flies’ energy expenditure by disrupting mitochondrial homeostasis, increasing lipolysis and by inducing hypoglycemia. Supportively, FoxO1 has been shown to play a pivotal role in regulating cellular responses to fasting, as it regulates genes involved in both glucose and lipid metabolism [47], suggesting a conserved mechanism. In addition, *foxo* OE in larvae alters animals’ feeding behavior and mimics the phenotypic effects of starvation [48], causing a complete developmental arrest due to decreased cell size and number. Our data further highlight the fact that foxo controls bioenergetic signaling cascades and stress response pathways by adjusting energy production and distribution in the peripheral tissues.

Since *foxo* OE mediated longevity is tightly affected by tissue-specificity and efficient inter-tissue communication [19], we also studied muscle- or cardiomyocyte-targeted *foxo* OE mediated effects. Muscle-tissues (thorax’s fibrillar flight muscles, heart tube’s myocardial cell layer, and intestine’s visceral muscle layer) are central in the regulation of metabolic flexibility and plasticity in the fly, while studies show that effective interorgan signaling instigated by muscle-tissue responses plays a major role in promoting healthy aging [16,49,50]. Our data showed that strong muscle- or cardiomyocyte-specific *foxo* OE is noxious for *Drosophila*’s physiology, probably due to intense muscle wasting, while moderate *foxo* OE delayed the onset of age-related phenotypes and increased healthspan. Accordingly, strong muscle-specific *foxo* OE in healthy mice reduces skeletal muscle mass and disturbs glycemic control [51], thereby supporting forkhead factors as targets to reverse muscle wasting during muscle atrophy caused by extreme fasting or systemic diseases [52]. On the other hand, moderate muscle-specific *foxo* OE in *Drosophila* flies was found to preserve muscle integrity and function during aging by modulating the accumulation of protein aggregates and nutrient-sensing mechanisms [16], thus supporting the whole organism’s homeostasis.

In addition, FOXO isoforms have been shown to have divergent functions in cardiac response to stress signals, and targeting FOXO proteins could inhibit or even reverse cardiac hypertrophy. More specifically, and in line with our findings, studies in mice show that FOXO1/FOXO3 activation decreases cardiomyocytes, while sustained FOXO3 activity leads to reversible heart atrophy [53,54,55]. Moreover, studies in *Drosophila* suggest that moderate heart-specific *foxo* OE is involved in the amelioration of cardiac age-associated function decline [35], and our study supports that this can also lead to increased longevity. Beyond metabolic modules, we found foxo to activate several cytoprotective modules and specifically the UPP components. Post-translation modifications that activate FOXO proteins have long been associated with the modulation of ALP, the deregulation of which plays a major role in proteostasis disruption that marks several age-related diseases, such as obesity, diabetes, cancer, and cardiovascular diseases [15]. FOXO-induced ALP upregulation, as a response to calorie restriction, has long been associated with reduced protein aggregation, delayed onset of functional decay, and enhanced longevity in both *C. elegans* and *D. melanogaster* [12]. On the other hand, although FOXOs also seem to modulate UPP by controlling ubiquitin ligases or proteasome composition, the FOXO-UPP axis’ contribution to lifespan regulation needs further investigation [15]. Our data suggest that either ubiquitous or tissue-specific *foxo* OE induces the upregulation of proteostatic modules in a dose-dependent manner. More specifically, although UPP upregulation was not always associated with *foxo* OE, increased UPP activity was a key feature of long-lived tissue-specific *foxo* overexpressing flies, further supporting the notion that the ability of the organism to maintain proteostasis correlates strongly with a delay in tissue aging. In support, it was shown that FOXO directly regulates the activity of 19S proteasome by upregulating the Rpn6 proteasome subunit, promoting resistance to various types of exogenous stressors [56,57]; also, FoxO1 was found to regulate both the expression and activity of the 20S proteasome by directly binding to the *β5* proteasome gene’s promoter [58]. Further investigation in our study using genetic or pharmacological interventions revealed that proteasome activation in *foxo* overexpressing *Drosophila* lines is Nrf2/cncC-mediated. It was shown previously that the functional crosstalk between stress-responsive elements, upon, e.g., starvation-like conditions, precisely orchestrates genome-wide changes to induce specific gene expression programs and balance aberrant cellular damage, thereby impacting lifespan [3,59]. Furthermore, apart from highlighting the coordinated action of foxo and Nrf2/cncC under stress conditions, our findings exemplify the detrimental effects of prolonged activation of stress sensors on an organism’s physiology and longevity [25].

Taken together, our findings suggest the differential dose- and tissue-dependent effects of foxo on longevity-associated signaling cascades, such as bioenergetic and proteostatic pathways. Sustained ubiquitous *foxo* upregulation exhausted energy expenditure, triggered proteostasis, in a coordinated action with Nrf2/cncC, and affected lifespan in a dose-dependent manner, supporting the notion that prolonged intense activation of stress sensors is not a favorable condition. On the other hand, depending on the metabolic profile of the targeted tissue, a specific dose of *foxo* OE improved flies’ healthspan by delaying the onset of age-related functional decline. Thus, defining that context-depended activity (i.e., dose and tissue) may be required to unleash FOXO’s beneficial potential as a strategy to prevent age-related diseases and improve life quality.

## Data Availability

The datasets generated and/or analyzed during the current study are available from the corresponding author on reasonable request.

## Supplementary Materials

The following are available online at www.mdpi.com/article/10.3390/cells10123577/s1. Supplementary Information file includes: Figure S1. Molecular characterization of *Drosophila* transgenic lines overexpressing *foxo*; Figure S2. Longitudinal muscle fibers integrity during aging and quantification of IIS modules’ protein levels expression; Figure S3. Ubiquitous *foxo* OE delays flies’ recovery following thermal stress; Figure S4. CLSM images of foxo cytoplasmic accumulation and translocation to the nucleus after muscle-specific *foxo* induction; Figure S5. CLSM images of foxo cytoplasmic accumulation and translocation to the nucleus after cardiomyocyte-specific *foxo* induction; Figure S6. Dose-dependent activation of proteostatic modules after tissue-specific *foxo* OE in *Drosophila* flies; Figure S7. *Nrf2/cncC-* and downstream proteasome-activation after sustained *foxo* OE is vital for transgenic flies’ survival; Figure S8. Combined *foxo* and *Nrf2/cncC* OE enhances proteasome activity; yet in the long term it is toxic; a list of *Drosophila* genes assayed in this study; a list of abbreviations; the graphical abstract legend; and Table S1. Summary of lifespan experiments and longevity statistics. Moreover, we provide full-length immunoblots images for each experiment shown in Figures and Supplementary Figures.

## References

[1] da Costa J.P., Vitorino R., Silva G.M., Vogel C., Duarte A.C., Rocha-Santos T. (2016). A synopsis on aging—Theories, mechanisms and future prospects. Ageing Res. Rev..

[2] Kennedy B.K., Berger S.L., Brunet A., Campisi J., Maria A., Epel E.S., Franceschi C., Lithgow G.J., Richard I. (2016). Aging: A Common Driver of Chronic Diseases and a Target for Novel Interventions. Cell.

[3] Martínez Corrales G., Alic N. (2020). Evolutionary Conservation of Transcription Factors Affecting Longevity. Trends Genet..

[4] Friedman D., Johnson T. (1988). A Mutation in the Age-1 Gene in *Caenorhabditis elegans* Lengthens Life and Reduces Hermaphrodite Fertility. Genetics.

[5] Johnson T.E. (2013). 25years after age-1: Genes, interventions and the revolution in aging research. Exp. Gerontol..

[6] Kaushik S., Cuervo A.M. (2015). Proteostasis and aging. Nat. Med..

[7] López-Otín C., Blasco M.A., Partridge L., Serrano M., Kroemer G. (2013). The Hallmarks of Aging. Cell.

[8] Klaips C.L., Jayaraj G.G., Hartl F.U. (2018). Pathways of Cellular Proteostasis in Aging and Disease. J. Cell Biol..

[9] Tsakiri E.N., Trougakos I.P. (2015). The amazing ubiquitin-proteasome system: Structural components and implication inaging. Int. Rev. Cell Mol. Biol..

[10] Hipp M.S., Kasturi P., Hartl F.U. (2019). The proteostasis network and its decline in ageing. Nat. Rev. Mol. Cell Biol..

[11] Webb A.E., Brunet A. (2014). FOXO transcription factors: Key regulators of cellular quality control. Trends Biochem. Sci..

[12] Martins R., Lithgow G.J., Link W. (2016). Long live FOXO: Unraveling the role of FOXO proteins in aging and longevity. Aging Cell.

[13] Eijkelenboom A., Burgering B.M.T. (2013). FOXOs: Signalling integrators for homeostasis maintenance. Nat. Rev. Mol. Cell Biol..

[14] Sen P., Shah P.P., Nativio R., Berger S.L. (2016). Epigenetic Mechanisms of Longevity and Aging. Cell.

[15] Cheng Z. (2019). The FoxO–Autophagy Axis in Health and Disease. Trends Endocrinol. Metab..

[16] Demontis F., Perrimon N. (2010). FOXO/4E-BP Signaling in *Drosophila* Muscles Regulates Organism-wide Proteostasis during Aging. Cell.

[17] Hwangbo D.S., Gersham B., Tu M., Palmer M. (2004). *Drosophila* dFOXO controls lifespan and regulates insulin signalling in brain and fat body. Nature.

[18] Giannakou M.E., Goss M., Juenger M.A., Ju M.A., Hafen E., Leevers S.J., Partridge L. (2004). Long-Lived *Drosophila* with Over- Expressed DFOXO in Adult Fat Body. Science.

[19] Poirier L., Shane A., Zheng J., Seroude L. (2008). Characterization of the *Drosophila* Gene-Switch System in Aging Studies: A Cautionary Tale. Aging Cell.

[20] Smith H.J., Sharma A., Mair W.B. (2020). Metabolic Communication and Healthy Aging: Where Should We Focus Our Energy?. Dev. Cell.

[21] Ugur B., Chen K., Bellen H.J. (2016). *Drosophila* Tools and Assays for the Study of Human Diseases. Dis. Model. Mech..

[22] Hales K.G., Korey C.A., Larracuente A.M., Roberts D.M. (2015). Genetics on the Fly: A Primer on the *Drosophila* Model System. Genetics.

[23] Trougakos I.P., Margaritis L.H. (1998). Immunolocalization of the Temporally “early” Secreted Major Structural Chorion Proteins, Dvs38 and Dvs36, in the Eggshell Layers and Regions of *Drosophila Virilis*. J. Struct. Biol..

[24] Tsakiri E.N., Sykiotis G.P., Papassideri I.S., Gorgoulis V.G., Bohmann D., Trougakos I.P. (2013). Differential Regulation of Proteasome Functionality in Reproductive vs. Somatic Tissues of *Drosophila* during Aging or Oxidative Stress. FASEB J..

[25] Tsakiri E., Gumeni S., Iliaki K., Benaki D., Sykiotis G.P., Gorgoulis V.G., Scorrano L., Trougakos I.P., Iliaki K.K., Tsakiri E.N. (2019). Hyperactivation of Nrf2 Increases Stress Tolerance at the Cost of Aging Acceleration Due to Metabolic Deregulation. Aging Cell.

[26] Manola M.S., Tsakiri E.N., Trougakos I.P. (2019). Alterations in Organismal Physiology, Impaired Stress Resistance, and Accelerated Aging in *Drosophila* Flies Adapted to Multigenerational Proteome Instability. Oxid. Med. Cell. Longev..

[27] Alayari N.N., Vogler G., Taghli-Lamallem O., Ocorr K., Bodmer R., Cammarato A. (2009). Fluorescent Labeling of *Drosophila* Heart Structures. J. Vis. Exp..

[28] Tsakiri E.N., Gaboriaud-Kolar N., Iliaki K.K., Tchoumtchoua J., Papanagnou E.-D., Chatzigeorgiou S., Tallas K.D., Mikros E., Halabalaki M., Skaltsounis A.-L. (2017). The Indirubin Derivative 6-Bromoindirubin-3′-Oxime Activates Proteostatic Modules, Reprograms Cellular Bioenergetic Pathways, and Exerts Antiaging Effects. Antioxid. Redox Signal..

[29] Ayrinhac A., Debat V., Gibert P., Kister A.G., Legout H., Moreteau B., Vergilino R., David J.R. (2004). Cold Adaptation in Geographical Populations of *Drosophila melanogaster*: Phenotypic Plasticity Is More Important than Genetic Variability. Funct. Ecol..

[30] Gumeni S., Evangelakou Z., Tsakiri E.N., Scorrano L., Trougakos I.P. (2019). Functional Wiring of Proteostatic and Mitostatic Modules Ensures Transient Organismal Survival during Imbalanced Mitochondrial Dynamics. Redox Biol..

[31] Barrio L., Dekanty A., Milán M. (2014). MicroRNA-Mediated Regulation of Dp53 in the *Drosophila* Fat Body Contributes to Metabolic Adaptation to Nutrient Deprivation. Cell Rep..

[32] Matsushita R., Nishimura T. (2020). Trehalose Metabolism Confers Developmental Robustness and Stability in *Drosophila* by Regulating Glucose Homeostasis. Commun. Biol..

[33] Grönke S., Mildner A., Fellert S., Tennagels N., Petry S., Müller G., Jäckle H., Kühnlein R.P. (2005). Brummer Lipase Is an Evolutionary Conserved Fat Storage Regulator in *Drosophila*. Cell Metab..

[34] Kim M.S., Pak Y.K., Jang P.G., Namkoong C., Choi Y.S., Won J.C., Kim K.S., Kim S.W., Kim H.S., Park J.Y. (2006). Role of hypothalamic Foxo1 in the regulation of food intake and energy homeostasis. Nat. Neurosci..

[35] Blice-Baum A.C., Kaushik G., Viswanathan M.C., Zambon A.C., Engler A.J., Bodmer R., Cammarato A. (2015). Overexpression of Foxo in the Heart Ameliorates Performance Decline through Enhanced UPS Processing in Aging *Drosophila*. Biophys. J..

[36] Tullet J.M.A., Hertweck M., An J.H., Baker J., Hwang J.Y., Liu S., Oliveira R.P., Baumeister R., Blackwell T.K. (2008). Direct Inhibition of the Longevity-Promoting Factor SKN-1 by Insulin-like Signaling in *C. elegans*. Cell.

[37] Zhang Z.D., Milman S., Lin J.-R., Wierbowski S., Yu H., Barzilai N., Gorbunova V., Ladiges W.C., Niedernhofer L.J., Suh Y. (2020). Genetics of Extreme Human Longevity to Guide Drug Discovery for Healthy Ageing. Nat. Metab..

[38] Alic N., Andrews T.D., Giannakou M.E., Papatheodorou I., Slack C., Hoddinott M.P., Cochemé H.M., Schuster E.F., Thornton J.M., Partridge L. (2011). Genome-wide DFOXO Targets and Topology of the Transcriptomic Response to Stress and Insulin Signalling. Mol. Syst. Biol..

[39] Wang Z., Yu T., Huang P. (2016). Post-translational modifications of FOXO family proteins (Review). Mol. Med. Rep..

[40] Gui T., Burgering B. (2021). FOXOs: Masters of the equilibrium. FEBS J..

[41] Hannenhalli S., Kaestner K.H. (2009). The Evolution of Fox Genes and Their Role in Development and Disease. Nat. Rev. Genet..

[42] Daitoku H., Kaneko Y., Yoshimochi K., Matsumoto K., Araoi S., Sakamaki J.I., Takahashi Y., Fukamizu A. (2016). Non-transcriptional Function of FOXO1/DAF-16 Contributes to Translesion DNA Synthesis. Mol. Cell Biol..

[43] Zhao Y., Yang J., Liao W., Liu X., Zhang H., Wang S., Wang D., Feng J., Yu L., Zhu W.G. (2012). Cytosolic FoxO1 is essential for the induction of autophagy and tumour suppressor activity. Nat. Cell Biol..

[44] van der Vos K.E., Coffer P.J. (2011). The extending network of FOXO transcriptional target genes. Antioxid. Redox Signal..

[45] Tsai W.-B., Chung Y.M., Takahashi Y., Xu Z., Hu M.C.T. (2008). Functional interaction between FOXO3a and ATM regulates DNA damage response. Nat. Cell Biol..

[46] Cao Y., Kamioka Y., Yokoi N., Kobayashi T., Hino O., Onodera M., Mochizuki N., Nakae J. (2006). Interaction of FoxO1 and TSC2 induces insulin resistance through activation of the mammalian target of rapamycin/p70 S6K pathway. J. Biol. Chem..

[47] Gross D.N., van den Heuvel A.P.J., Birnbaum M.J. (2008). The Role of FoxO in the Regulation of Metabolism. Oncogene.

[48] Kramer J.M., Davidge J.T., Lockyer J.M., Staveley B.E. (2003). Expression of *Drosophila* FOXO Regulates Growth and Can Phenocopy Starvation. BMC Dev. Biol..

[49] Smith R.L., Soeters M.R., Wüst R.C.I., Houtkooper R.H. (2018). Metabolic Flexibility as an Adaptation to Energy Resources and Requirements in Health and Disease. Endocr. Rev..

[50] Argilés J.M., Campos N., Lopez-Pedrosa J.M., Rueda R., Rodriguez-Mañas L. (2016). Skeletal Muscle Regulates Metabolism via Interorgan Crosstalk: Roles in Health and Disease. J. Am. Med. Dir. Assoc..

[51] Kamei Y., Miura S., Suzuki M., Kai Y., Mizukami J., Taniguchi T., Mochida K., Hata T., Matsuda J., Aburatani H. (2004). Skeletal muscle FOXO1 (FKHR) transgenic mice have less skeletal muscle mass, down-regulated Type I (slow twitch/red muscle) fiber genes, and impaired glycemic control. J. Biol. Chem..

[52] Sandri M., Barberi L., Bijlsma A.Y., Blaauw B., Dyar K.A., Milan G., Mammucari C., Meskers C.G.M., Pallafacchina G., Paoli A. (2013). Signalling Pathways Regulating Muscle Mass in Ageing Skeletal Muscle. the Role of the IGF1-Akt-MTOR-FoxO Pathway. Biogerontology.

[53] Hariharan N., Ikeda Y., Hong C., Alcendor R.R., Usui S., Gao S., Maejima Y., Sadoshima J. (2013). Autophagy Plays an Essential Role in Mediating Regression of Hypertrophy during Unloading of the Heart. PLoS ONE.

[54] Cao D.J., Jiang N., Blagg A., Johnstone J.L., Gondalia R., Oh M., Luo X., Yang K., Shelton J.M., Rothermel B.A. (2013). Mechanical Unloading Activates FoxO3 to Trigger Bnip3-Dependent Cardiomyocyte Atrophy. J. Am. Heart Assoc..

[55] Schips T.G., Wietelmann A., Höhn K., Schimanski S., Walther P., Braun T., Wirth T., Maier H.J. (2011). FoxO3 Induces Reversible Cardiac Atrophy and Autophagy in a Transgenic Mouse Model. Cardiovasc. Res..

[56] Vilchez D., Morantte I., Liu Z., Douglas P.M., Merkwirth C., Rodrigues A.P.C., Manning G., Dillin A. (2012). RPN-6 Determines *C. elegans* Longevity under Proteotoxic Stress Conditions. Nature.

[57] Vilchez D., Boyer L., Morantte I., Lutz M., Merkwirth C., Joyce D., Spencer B., Page L., Masliah E., Travis Berggren W. (2012). Increased Proteasome Activity in Human Embryonic Stem Cells Is Regulated by PSMD11. Nature.

[58] Kapetanou M., Nespital T., Tain L.S., Pahl A., Partridge L., Gonos E.S. (2021). FoxO1 Is a Novel Regulator of 20S Proteasome Subunits Expression and Activity. Front. Cell Dev. Biol..

[59] Kocaturk N.M., Gozuacik D. (2018). Crosstalk Between Mammalian Autophagy and the Ubiquitin-Proteasome System. Front. Cell Dev. Biol..

