# Supplementary material for: Differential Dose- and Tissue-Dependent Effects of foxo on Aging, Metabolic and Proteostatic Pathways
Source: Cells. 2021 Dec 18;10(12):3577. doi: 10.3390/cells10123577 (PMC8700554; doi:10.3390/cells10123577)
Supplement: Supplementary file 1 [file cells-10-03577-s001.zip › cells-1406066-supplement materials/cells-1406066_Suppl_Information.pdf]

Article

Differential dose- and tissue-dependent effects of foxo on aging, metabolic and proteostatic pathways

Maria S. Manola, Sentiljana Gumeni and Ioannis P. Trougakos*

Department of Cell Biology and Biophysics, Faculty of Biology, National and Kapodistrian University of Athens, Panepistimiopolis, 15784, Athens, Greece; mmanola@biol.uoa.gr (M.S.M.), sgumeni@biol.uoa.gr (S.G.)
* **Correspondence**: itrougakos@biol.uoa.gr (I.P.T.), Tel: +30-210-7274555

SUPPLEMENTARY INFORMATION

Supplementary Figures

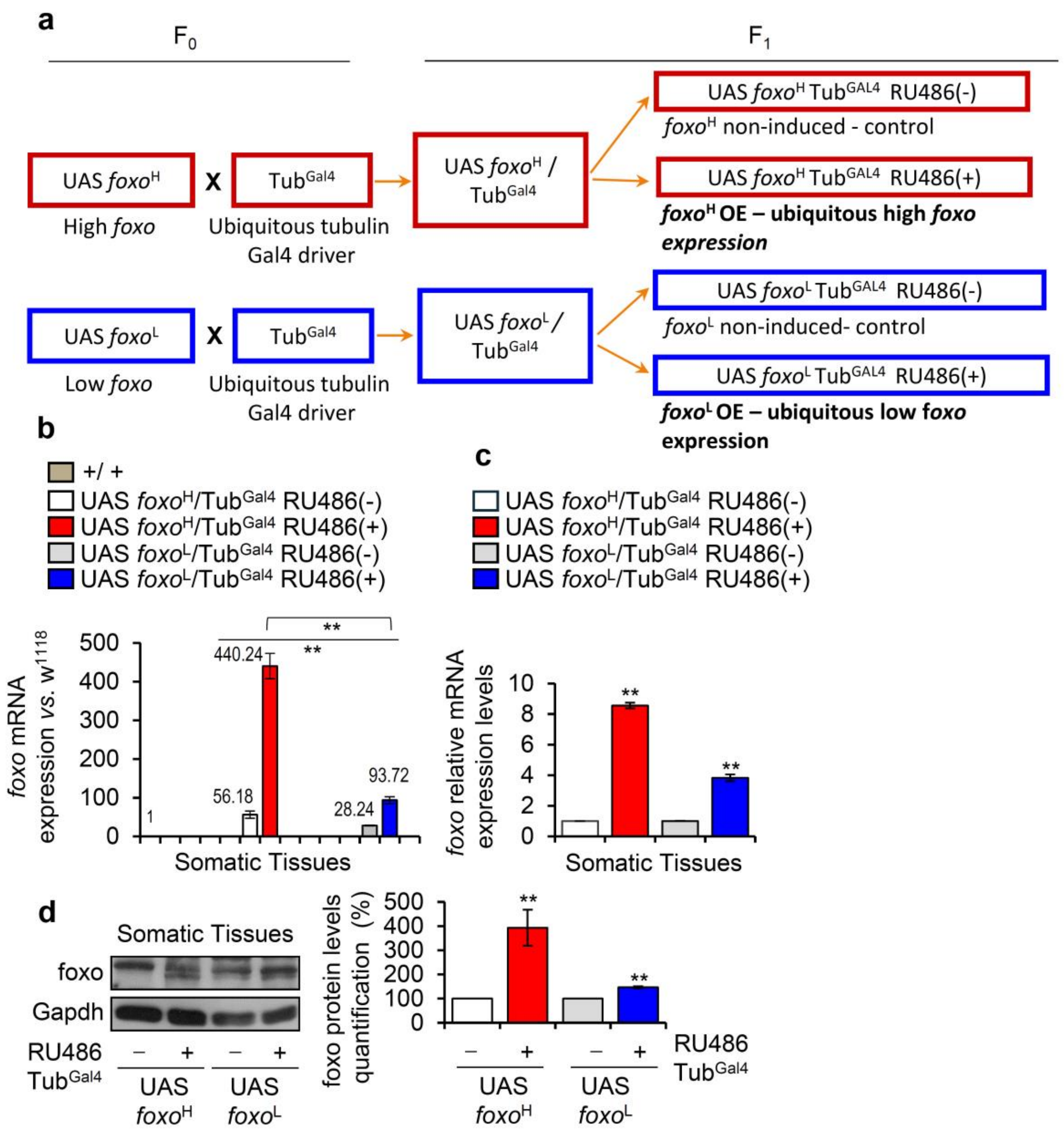

Figure S1. Molecular characterization of *Drosophila* transgenic lines overexpressing *foxo*. (**a**) Schematic illustration of our crossbreeding design to achieve ubiquitous *foxo*^{H} or *foxo*^{L} OE in somatic tissues of *Drosophila melanogaster* flies. Transgenic flies were cultured with 320 μM RU486 [RU486(+)] to induce transgene(s) expression; untreated flies [RU486(-)] were used as controls. (**b**, **c**) Relative *foxo* mRNA expression levels in *foxo* overexpressing flies' somatic tissues 7 days post-transgene induction *vs*. the w^{1118} (wild type) line (**b**) or transgenic not induced flies (**c**). *RpL32* gene expression levels were used as input reference; gene expression was plotted *vs*. the respective control set to 1. (**d**) Immunoblotting analysis of foxo protein expression levels (left panel) and quantification (%) (right panel) in transgenic flies' somatic tissues 7 days post transgene induction. Control values were set to 100%. Gapdh probing was used as a loading control. Bars, ± SD; n ≥ 2, 20 flies were analyzed per experimental repeat. Statistical significance was measured with unpaired *t*-test unpaired t-test, ** $p < 0.01$.

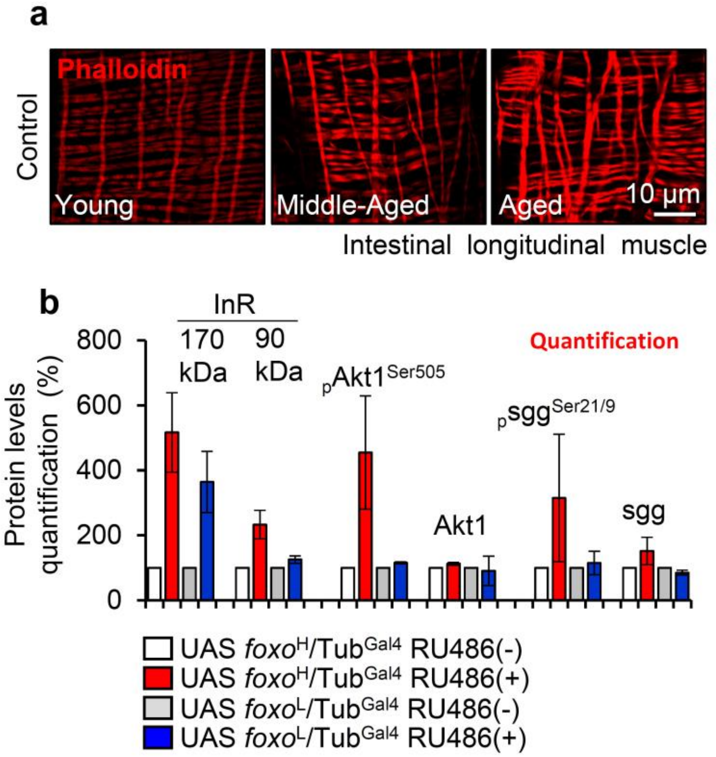

Figure S2. Longitudinal muscle fibers integrity during aging and quantification of IIS modules' protein levels expression. (**a**) Representative CLSM images of intestinal longitudinal muscle fibers' actin filaments (Phalloidin staining) of female young, middle-aged, and aged w^{1118} wild type (control) flies, n ≥ 3; 10 female flies were analyzed per each experimental repeat. (**b**) Relative (% *vs*. controls) quantification of indicated proteins levels at immunoblots shown at Figure 2d. Control values were set to 100% and Gapdh was used as a loading control. Bars, ± SD; n ≥ 2. In (**b)**, statistical significance was measured with unpaired *t*-test.

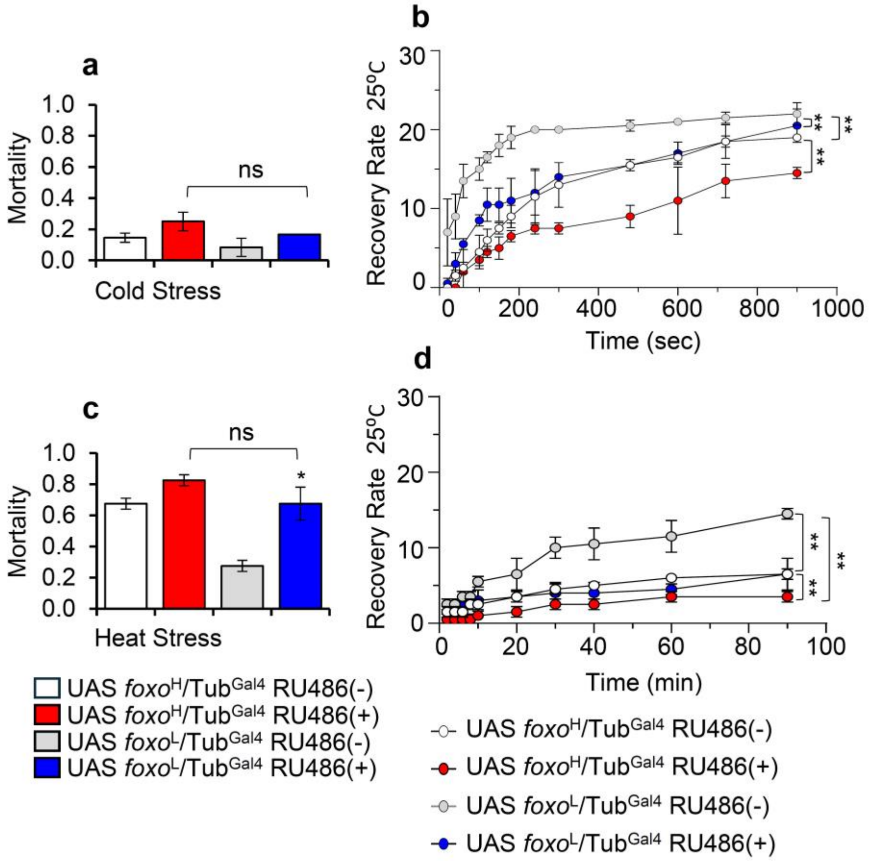

Figure S3. Ubiquitous *foxo* OE delays flies' recovery following thermal stress. (**a**) Mortality and, (**b**) recovery rates of shown *foxo* transgenic flies following cold sensitivity assay (see, Materials and Methods). (**c**) Mortality and (**d**) recovery rates of *foxo* overexpressing flies following transient exposure to heat stress (see, Materials and Methods). Bars, ± SD; n = 2, 20 flies were analyzed per experimental repeat. In (**a**, **c)** statistical significance was calculated using the unpaired t-test; ns, non-significant. In (**a, c**), statistical significance was measured with unpaired *t*-test, ns, non-significant. In (**b**, **d**) a two-way ANOVA test followed by Tukey's multiple comparisons test was performed to determine statistical significance, ** $p < 0.01$.

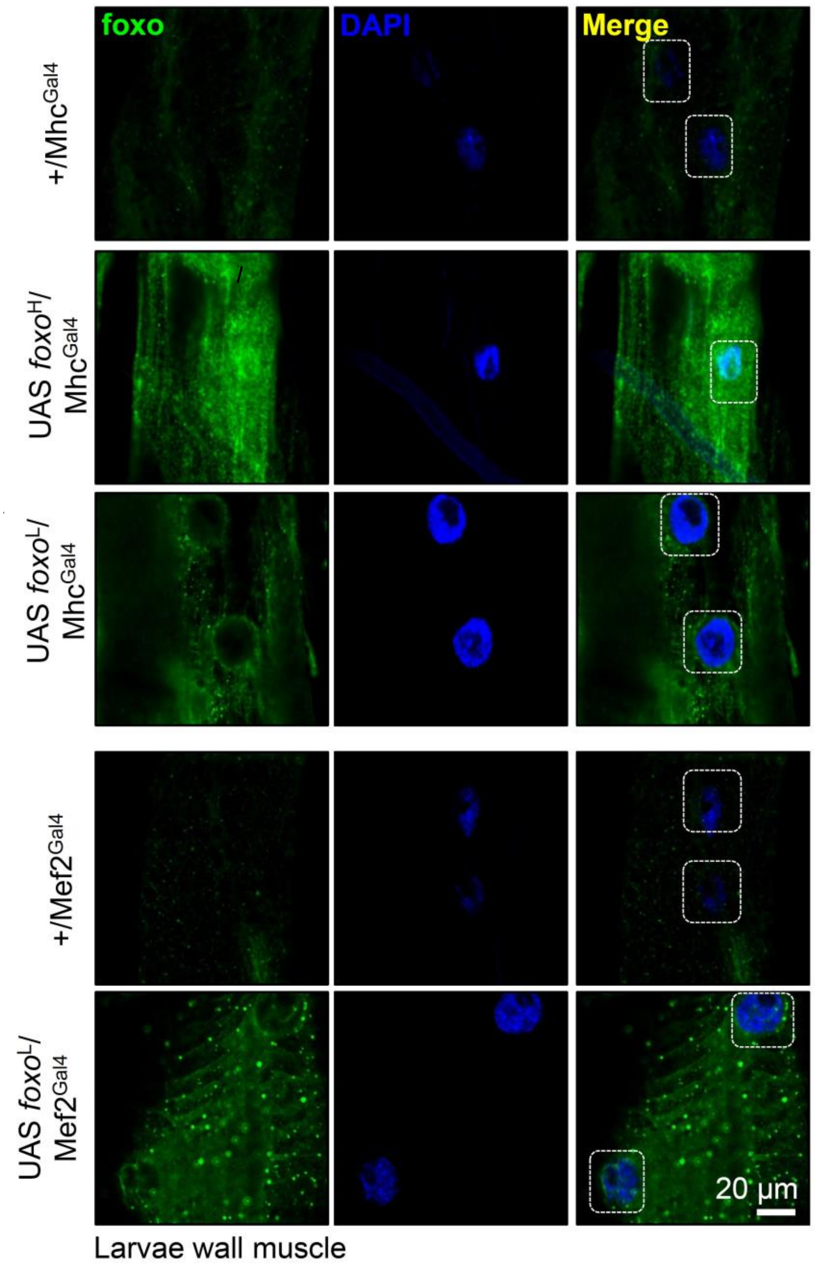

Figure S4. CLSM images of foxo cytoplasmic accumulation and translocation to the nucleus after muscle-specific *foxo* induction. Representative CLSM images of third instar larvae body wall muscle fibers after targeted *foxo* OE in muscles (using moderate Mhc^{Gal4} or strong $Mef2^{Gal4}$ drivers); samples were stained with an anti-FOXO antibody and counterstained with DAPI. n ≥ 3, 10 flies were analyzed per experimental repeat.

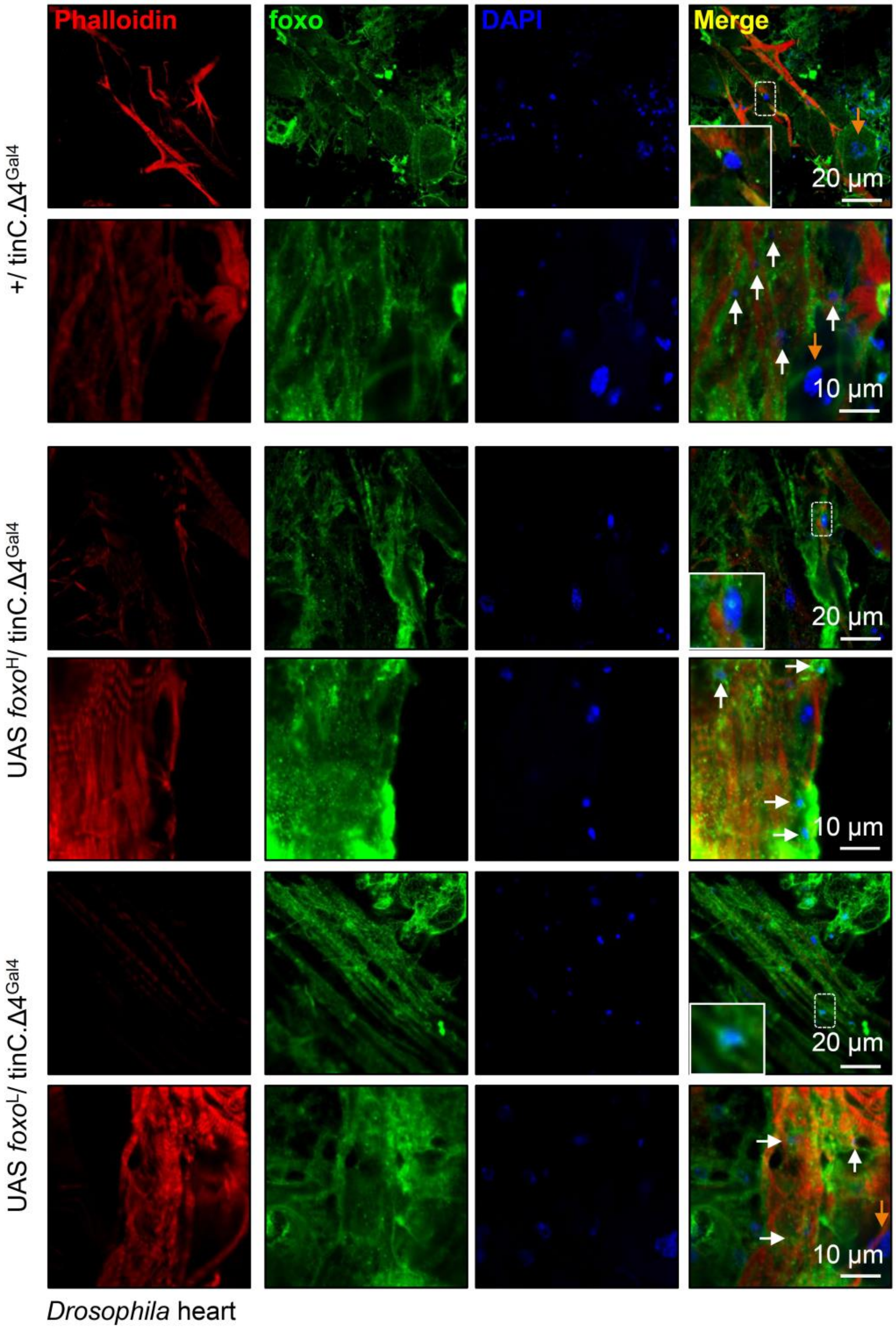

Figure S5. CLSM images of foxo cytoplasmic accumulation and translocation to the nucleus after cardiomyocyte-specific *foxo* induction. Representative CLSM images of intact young *Drosophila* hearts following $foxo^{H}$ or $foxo^{L}$ cardiomyocyte-specific (tinC.$\Delta 4^{Gal4}$) OE; samples were stained with an anti-FOXO antibody and counterstained with DAPI to visualize nuclei. White arrows show cardiomyocytes' nuclei and orange arrows show pericardial cells' nuclei. n ≥ 3, 10 flies were analyzed per experimental repeat.

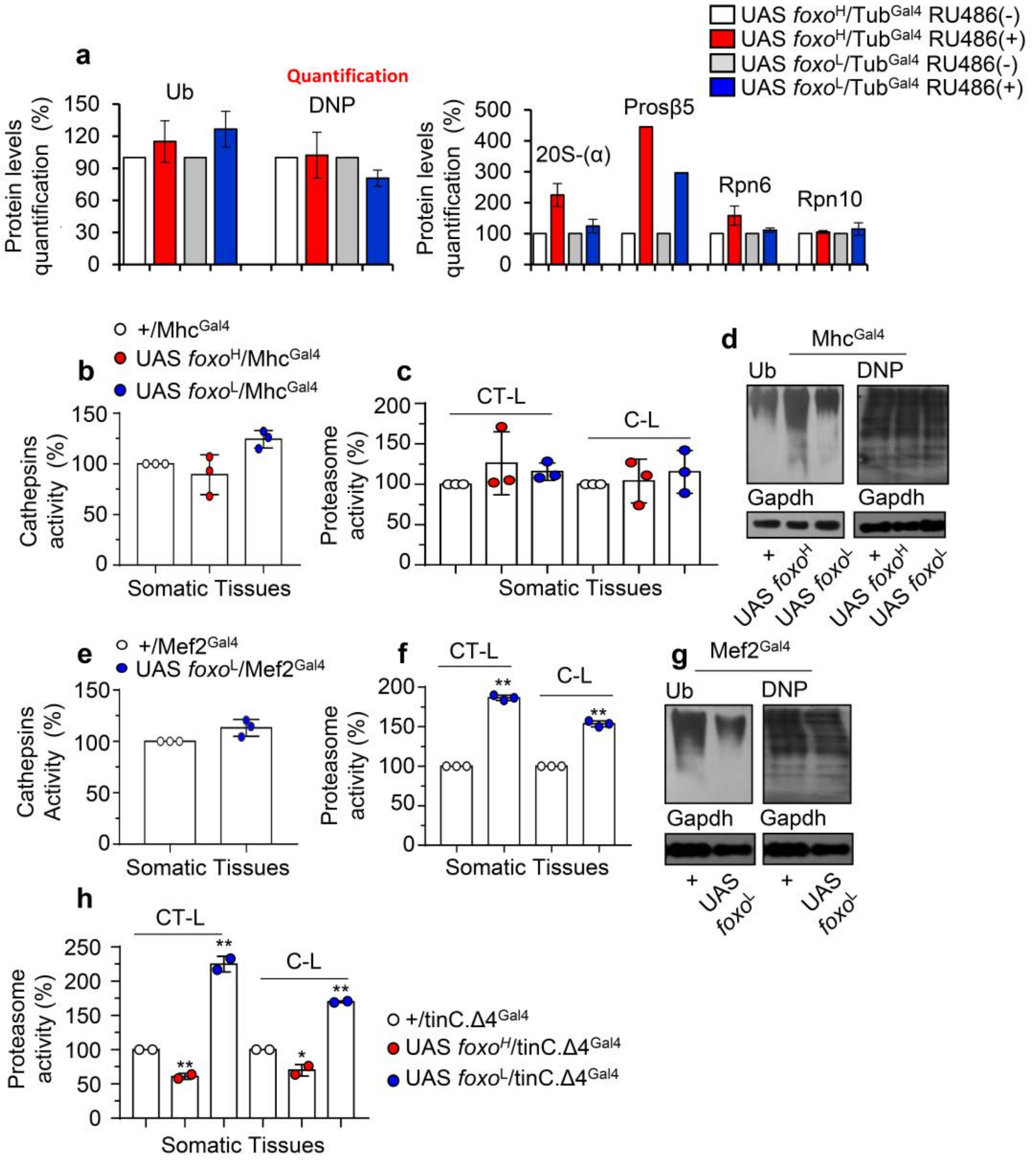

Figure S6. Dose-dependent activation of proteostatic modules after tissue-specific *foxo* OE in *Drosophila* flies. (**a**) Relative (%) *vs*. respective control quantification of immunoblots shown at Figure 6f. (**b**) Cathepsins activity (%), (**c**) 26S CT-L and C-L proteasome activity (%) (bars are as in **b**), and (**d**) immunoblots of total ubiquitinated (Ub) and carbonylated (DNP) protein levels in somatic tissues' lysates of young *Drosophila* flies after moderate muscle-specific (Mhc^{Gal4}) induction of $foxo^{H}$ or $foxo^{L}$ transgenes. (**e**) Cathepsins activity (%), (**f**) 26S CT-L and C-L proteasome activity (%) (bars are as in **e**) and, (**g**) immunoblots of total ubiquitinated (Ub) and carbonylated (DNP) protein levels in somatic tissues' lysates of young *Drosophila* flies after strong muscle-specific ($Mef2^{Gal4}$) induction of $foxo^{L}$. (**h**) 26S CT-L and C-L proteasome activity (%) in somatic tissues' lysates of *Drosophila* flies following heart-specific ($tinC.\Delta4^{Gal4}$) OE of $foxo^{H}$ or $foxo^{L}$ transgenes. In (**a-c, e**, **f**, **h**) control values were set to 100%. In (**a, d**, **g**) Gapdh was used as a loading control. Bars, ± SD; n ≥ 2, 10 flies were analyzed per experimental repeat. In (**a**), statistical significance was measured with unpaired *t*-test unpaired t-test. In (**b**, **c**, **e**, **f**, **h)** significant differences were calculated with a two-way ANOVA test followed by Tukey's multiple comparisons test, * $p < 0.05$, ** $p < 0.01$.

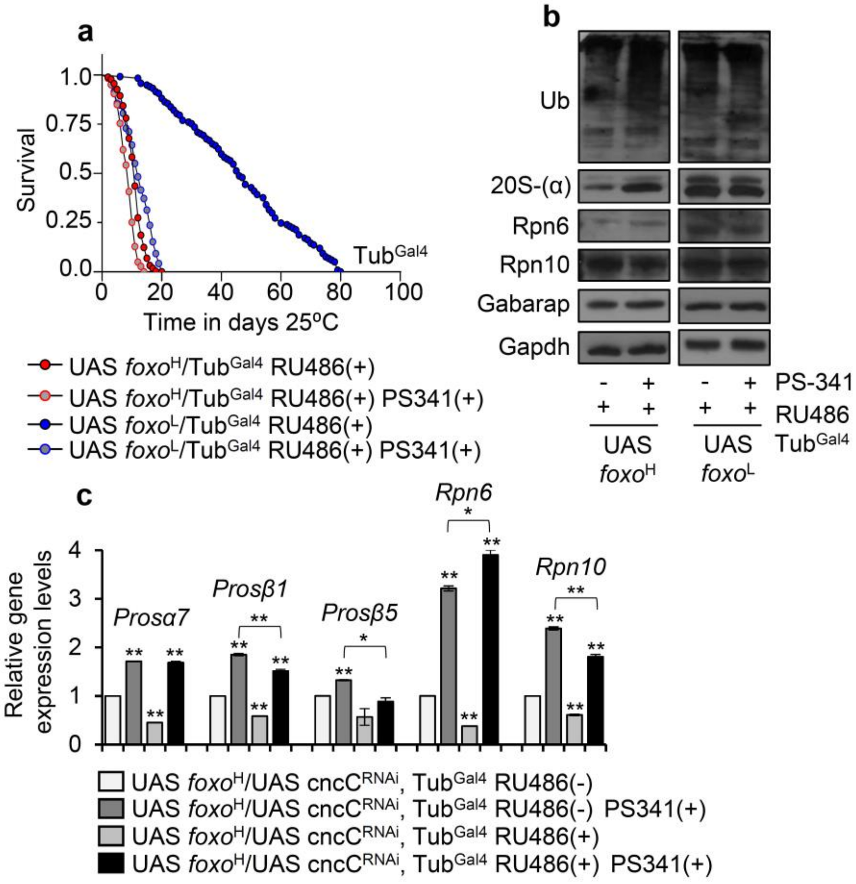

Figure S7. *Nrf2/cncC-* and downstream proteasome-activation after sustained *foxo* OE is vital for transgenic flies' survival. (**a**) Longevity curves of *foxo* overexpressing transgenic flies cultured (or not) with the proteasome inhibitor PS341 (5 μM) for 3 days. Longevity statistics are reported in Table S1. (**b**) Immunoblots of somatic tissues' protein lysates isolated from *foxo* overexpressing flies exposed (or not) to 5 μM PS341 for 3 days; antibodies used were against total ubiquitinated (Ub) proteins, proteasome [20S-(*α*), Rpn6, Rpn10] subunits and the autophagy-related Gabarap protein. Gapdh was used as loading control. (**c**) Relative expression levels of proteasome (*Prosα7*, *Prosb1*, *Prosβ5*, *Rpn6*, *Rpn10*) genes. Gene expression was plotted *vs*. shown controls set to 1. The *RpL32* gene expression was used as input reference. Bars, ± SD; n ≥ 2, > 10 flies were analyzed per experimental repeat. Statistical significance was calculated with unpaired t-test, * $p < 0.05$, ** $p < 0.01$.

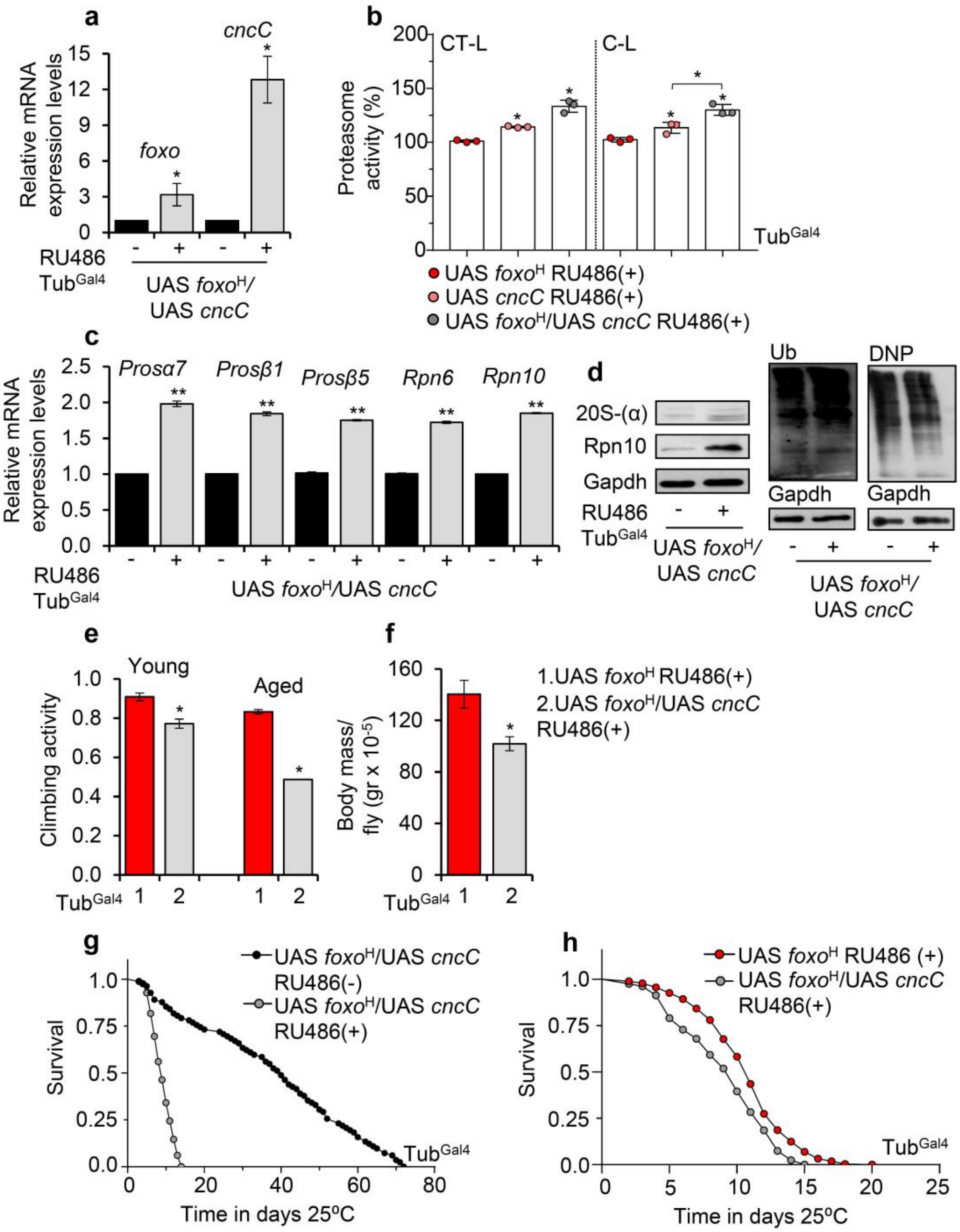

Figure S8. Combined *foxo* and *Nrf2*/*cncC* OE enhances proteasome activity; yet in the long term it is toxic. (**a**) Relative expression levels of *foxo* and *Nrf2*/*cncC* genes in *foxo*^{H}/*cncC Drosophila* transgenic flies. (**b**) 26S CT-L and C-L proteasome activity (%) in somatic tissues of the shown transgenic lines; control values were set to 100%. (**c**) Relative expression levels of proteasome (*Prosα7*, *Prosβ1*, *Prosβ5*, *Rpn6*, *Rpn10*) genes in *foxo^{H}*/*cncC* flies *vs.* controls. (**d**) Immunoblotting analysis in protein lysates isolated from *foxo^{H}*/*cncC* flies' somatic tissues using antibodies against proteasomal subunits [20S-(α) and Rpn10], and total ubiquitinated (Ub) or carbonylated (DNP) proteins. Gapdh was used as a loading control. (**e**) Neuromuscular degeneration (climbing activity) during aging in shown transgenic flies. (**f**) Body mass per fly (gr × 10^{-5}) of *foxo^{H}*/*cncC vs. foxo*^{H} overexpressing transgenic flies. (**g**) Longevity curves of *foxo^{H}*/*cncC* flies *vs.* non-induced controls [RU486(-)] or (**h**) as compared to *foxo*^{H} flies. Statistics of longevity assays are reported in Table S1. In (**a-d**, **f**) experiments were performed in young flies 7

days post the transgenes' induction. In (**a**, **c**) gene expression was plotted *vs.* controls set to 1; the *RpL32* gene expression was used as input reference. Bars, ± SD; n ≥ 2, > 10 flies were analyzed per experimental repeat. In (**a**, **c**, **e**, **f**) statistical significance was calculated using the unpaired t-test, * $p < 0.05$, ** $p < 0.01$. In (**b**), a two-way ANOVA test followed by Tukey's multiple comparisons test was performed to determine statistical significance, * $p < 0.05$.

List of genes

Akt1 (Akt kinase, FBgn0010379, CG4006); *Atg8a* (autophagy-related 8a, FBgn0052672, CG32672); *blw* (bellwether, also known as *ATP5A* or *ATPsyn-α*, FBgn0011211, CG6312); *bmm* (also known as *ATGL*, brummer lipase, FBgn0036449, CG5295); *cncC* (cap-n-collar isoform-C, FBgn0262975, CG43286); *Drp1* (dynamin related protein 1, FBgn0026479, CG3210); *foxo* (forkhead box, subgroup O, FBgn0038197, CG3143); *G6P* (glucose-6-phosphatase, FBgn0031463, CG15400); *GlyP* (glycogen phosphorylase, FBgn0004507, CG7254); *GlyS* (glycogen synthase, FBgn0266064, CG6904); *HDAC6* (histone deacetylase 6, FBgn0026428, CG6170); *Ilp2* (insulin-like peptide 2, FBgn0036046, CG8167); *Ilp6* (insulin-like peptide 6, FBgn0044047, CG14049); *Impl2* (ecdysone-inducible gene L2, FBgn001257, CG15009); *InR* (insulin-like receptor, FBgn0283499, CG18402); *Marf* (mitochondrial assembly regulatory factor, FBgn0029870, CG3869); *srl* (also known as *PGC-1*, spargel, FBgn0037248, CG9809); *Prosα7* (proteasome α7 subunit, FBgn0023175, CG1519); *Prosβ1* (proteasome β1 subunit, FBgn0010590, CG8392); *Prosβ5* (Proteasome β5 subunit, FBgn0029134, CG12323); *ref(2)P* (p62, refractory to sigma P, FBgn0003231, CG10360); *RpL32* (also known as *Rp49*, ribosomal protein L32, FBgn0002626, CG7939); *Rpn10* (regulatory particle non-ATPase 10, FBgn0015283, CG7619); *Rpn6* (regulatory particle non-ATPase 6, FBgn0028689, CG10149); *TFAM* (mitochondrial transcription factor A, FBgn0038805, CG4217).

List of abbreviations

Autophagy-lysosome pathway, ALP; cap'n'collar isoform C, cncC; caspase-like activity, C-L; chymotrypsin-like activity, CT-L; forkhead box O, FoxO; insulin/insulin-like growth factor signaling, IIS; nuclear factor erythroid 2 like 2, Nrf2; overexpression, OE; proteostasis network, PN; ubiquitin-proteasome pathway, UPP.

Graphical Abstract

foxo transcription factor acts as a cellular stress sensor and is a downstream target of the IIS. Upon fasting, foxo translocates to the nucleus, where it activates the transcription of (direct or indirect) longevity-associated gene expression programs. In this study, we induced ubiquitous, or tissue targeted OE of *foxo* in *Drosophila's* somatic or muscle-, heart-tissues, respectively. We used two transgenic lines that express distinct levels of *foxo* [i.e., high ($foxo^{H}$) or low ($foxo^{L}$) *foxo* expression levels]. Our data suggest that high, ubiquitous *foxo* OE deregulates proteostatic pathways and alters major metabolic signaling cascades, increasing sensitivity to stress, and ultimately leading to premature aging. Notably, there is a defined dosage of low tissue-specific *foxo* OE that promotes tissue integrity and overall homeostasis resulting in enhanced health-/life-span.
