# Supplementary material for: Differential Dose- and Tissue-Dependent Effects of foxo on Aging, Metabolic and Proteostatic Pathways
Source: Cells. 2021 Dec 18;10(12):3577. doi: 10.3390/cells10123577 (PMC8700554; doi:10.3390/cells10123577)
Supplement: Supplementary file 1 [file cells-10-03577-s001.zip › cells-1406066-supplement materials/cells-1406066_Suppl_Table_S1.pdf]

Table S1. Summary of lifespan experiments and longevity statistics.

								Log Rank Value				
**Fig. 2a**	**Sample**	**Mean Lifespan (LF) +/- s.e.m. (Days)**		**Median Lifespan +/- s.e.m. (Days)**		**% Median LF vs. control**	**Max (Days)**	**UAS *foxo* ^{H}/Tub^{Gal4} RU486(-)**	**UAS *foxo* ^{H}/Tub^{Gal4} RU486(+)**	**UAS *foxo* ^{L}/Tub^{Gal4} RU486(-)**	**UAS *foxo* ^{L}/Tub^{Gal4} RU486(+)**	**Total Animals Died/Total**
	UAS *foxo* ^{H}/Tub^{Gal4} RU486(-)	52.50	1.64	58.00	1.40	100.00	78.00		0.00	0.00	0.53	110/110
	UAS *foxo* ^{H}/Tub^{Gal4} RU486(+)	10.70	0.20	11.00	0.21	18.97	20.00	0.00		0.00	0.00	273/273
	UAS *foxo* ^{L}/Tub^{Gal4} RU486(-)	66.90	2.28	68.00	3.16	117.24	86.00	0.00	0.00		0.00	40/40
	UAS *foxo* ^{L}/Tub^{Gal4} RU486(+)	46.20	1.82	46.00	1.93	79.31	80.00	0.53	0.00	0.00		117/117

								Log Rank Value			
**Fig. 3d**	**Sample**	**Mean Lifespan (LF) +/- s.e.m. (Days)**		**Median Lifespan +/- s.e.m. (Days)**		**% Median LF vs. control**	**Max (Days)**	**UAS *foxo* ^{H}/Tub^{Gal4} RU486(+)**	**UAS *foxo* ^{H}/Tub^{Gal4} RU486(+) PR**	**UAS *foxo* ^{H}/Tub^{Gal4} RU486(+)**	**Total Animals Died/Total**
	UAS *foxo* ^{H}/Tub^{Gal4} RU486(+)	10.70	0.20	11.00	0.21	100.00	20.00		0.18	0.00	273/273
	UAS *foxo* ^{H}/Tub^{Gal4} RU486(+) PR	10.49	0.22	10.00	0.35	90.91	16.00	0.18		0.00	193/193
	UAS *foxo* ^{H}/Tub^{Gal4} RU486(+) CR	6.80	0.24	7.00	0.34	63.64	12.00	0.00	0.00		118/118

								Log Rank Value			
**Fig. 3e**	**Sample**	**Mean Lifespan (LF) +/- s.e.m. (Days)**		**Median Lifespan +/- s.e.m. (Days)**		**% Median LF vs. control**	**Max (Days)**	**UAS *foxo* ^{L}/Tub^{Gal4} RU486(+)**	**UAS *foxo* ^{L}/Tub^{Gal4} RU486(+) PR**	**UAS *foxo* ^{L} /Tub^{Gal4} RU486(+)**	**Total Animals Died/Total**
	UAS *foxo* ^{L}/Tub^{Gal4} RU486(+)	46.20	1.82	46.00	1.93	100.00	80.00		0.73	0.00	117/117
	UAS *foxo* ^{L}/Tub^{Gal4} RU486(+) PR	49.18	1.48	50.00	2.28	108.70	78.00	0.73		0.00	120/120
	UAS *foxo* ^{L} /Tub^{Gal4} RU486(+) CR	25.13	0.84	26.00	1.17	56.52	42.00	0.00	0.00		120/120

								Log Rank Value				
**Fig. 3f**	**Sample**	**Mean Lifespan (LF) +/- s.e.m. (Days)**		**Median Lifespan +/- s.e.m. (Days)**		**% Median LF vs. control**	**Max (Days)**	**UAS *foxo* ^{H}/Tub^{Gal4} RU486(+)**	**UAS *foxo* ^{H}/Tub^{Gal4} RU486(+) SS**	**UAS *foxo* ^{L}/Tub^{Gal4} RU486(+)**	**UAS *foxo* ^{L}/Tub^{Gal4} RU486(+) SS**	**Total Animals Died/Total**
	UAS *foxo* ^{H}/Tub^{Gal4} RU486(+)	10.77	0.20	11.00	0.21	100.00	20.00		0.86	0.00	0.00	273/273
	UAS *foxo* ^{H}/Tub^{Gal4} RU486(+) SS	10.26	0.38	11.00	0.96	100.00	16.00	0.86		0.00	0.00	105/105
	UAS *foxo* ^{L}/Tub^{Gal4} RU486(+)	46.23	1.82	46.00	1.93	418.18	80.00	0.00	0.00		0.00	117/117
	UAS *foxo* ^{L}/Tub^{Gal4} RU486(+) SS	54.43	1.59	54.00	1.83	490.91	85.00	0.00	0.00	0.00		120/120

								Log Rank P Value			
**Fig. 4b**	**Sample**	**Mean Lifespan (LF) +/- s.e.m. (Days)**		**Median Lifespan +/- s.e.m. (Days)**		**% Median LF vs. control**	**Max (Days)**	**+/MhcGal4**	**UAS *foxo* ^{H} / Mhc^{Gal4}**	**UAS *foxo* ^{L} / Mhc^{Gal4}**	**Total Animals Died/Total**
	+/MhcGal4	58.738	0.943	58.000	1.080	100.00	8.00		0.00	0.00	229/229
	UAS *foxo* ^{H} / Mhc^{Gal4}	49.695	0.997	48.000	1.449	82.76	91.00	0.00		0.00	210/210
	UAS *foxo* ^{L} / Mhc^{Gal4}	65.995	1.329	66.000	2.079	113.79	102.00	0.00	0.00		197/197

								Log Rank P Value		
**Fig. 4f**	**Sample**	**Mean Lifespan (LF) +/- s.e.m. (Days)**		**Median Lifespan +/- s.e.m. (Days)**		**% Median LF vs. control**	**Max (Days)**	**+/Mef2Gal4**	**UAS *foxo* ^{L}/ Mef2^{Gal4}**	**Total Animals Died/Total**
	+/Mef2Gal4	57.48	1.11	59.00	1.62	100.00	80.00		0.00	197/197
	UAS *foxo* ^{L}/ Mef2^{Gal4}	70.58	1.58	74.00	2.52	125.42	102.00	0.00		175/175

								Log Rank P Value			
**Fig. 5c**	**Sample**	**Mean Lifespan (LF) +/- s.e.m. (Days)**		**Median Lifespan +/- s.e.m. (Days)**		**% Median LF vs. control**	**Max (Days)**	**+/tinC.Δ4^{Gal4}**	**UAS *foxo* ^{H}/tinC.Δ4^{Gal4}**	**UAS *foxo* ^{L}/tinC.Δ4^{Gal4}**	**Total Animals Died/Total**
	+/tinC.Δ4^{Gal4}	53.978	1.534	52.000	2.098	100.00	81.00		0.00	0.00	138/138
	UAS *foxo* ^{H}/tinC.Δ4^{Gal4}	22.710	1.736	19.000	4.000	36.54	58.00	0.00		0.00	100/100
	UAS *foxo* ^{L}/tinC.Δ4^{Gal4}	56.156	2.108	60.000	2.274	115.38	85.00	0.00	0.00		122/122

								Log Rank P Value		
**Fig. 7g**	**Sample**	**Mean Lifespan (LF) +/- s.e.m. (Days)**		**Median Lifespan +/- s.e.m. (Days)**		**% Median LF vs. control**	**Max (Days)**	**UAS *foxo* ^{H}/UAS *cncC* ^{RNAi} /Tub^{Gal4} RU486(-)**	**UAS *foxo* ^{H}/UAS *cncC* ^{RNAi} /Tub^{Gal4} RU486(+)**	**Total Animals Died/Total**
	UAS *foxo* ^{H}/UAS *cncC* ^{RNAi}/Tub^{Gal4} RU486(-)	36.200	2.561	53.000	2.981	100	79		0.00	120/120
	UAS *foxo* ^{H}/UAS *cncC* ^{RNAi}/Tub^{Gal4}RU486(+)	20.067	0.604	8.000	0.381	15.09433962	16	0.00		128/130

								Log Rank Value		
**Fig. 7h**	**Sample**	**Mean Lifespan (LF) +/- s.e.m. (Days)**		**Median Lifespan +/- s.e.m. (Days)**		**% Median LF vs. control**	**Max (Days)**	**UAS *foxo* ^{H}/ Tub^{Gal4} RU486(+)**	**UAS *foxo* ^{H}/UAS *cncC* ^{RNAi}/Tub^{Gal4} RU486(+)**	**Total Animals Died/Total**
	UAS *foxo* ^{H}/ Tub^{Gal4} RU486(+)	8.728	0.210	11.000	0.170	100	20		0.00	273/273
	UAS *foxo* ^{H}/UAS *cncC* ^{RNAi}/Tub^{Gal4} RU486(+)	20.067	0.604	8.000	0.381	72.72727273	16	0.00		128/130

								Log Rank Value				
**Suppl. Fig. S7a**	**Sample**	**Mean Lifespan (LF) +/- s.e.m. (Days)**		**Median Lifespan +/- s.e.m. (Days)**		**% Median LF vs. control**	**Max (Days)**	**UAS *foxo* ^{H}/Tub^{Gal4} RU486(+)**	**UAS *foxo* ^{H}/Tub^{Gal4} RU486(+) PS341 5 μM**	**UAS *foxo* ^{L}/Tub^{Gal4} RU486(+)**	**UAS *foxo* ^{L}/Tub^{Gal4} RU486(+) PS341 5 μM**	**Total Animals Died/Total**
	UAS *foxo* ^{H}/Tub^{Gal4} RU486(+)	10.77	0.20	11.00	0.21	100.00	20.00		0.00	0.00	0.00	273/273
	UAS *foxo* ^{H}/Tub^{Gal4} RU486(+) PS341 5 μM	8.46	0.21	9.00	0.35	81.82	14.00	0.00		0.00	0.00	179/179
	UAS *foxo* ^{L}/Tub^{Gal4} RU486(+)	45.86	1.86	46.00	3.833	418.18	80.00	0.00	0.00		0.00	117/117
	UAS *foxo* ^{L}/Tub^{Gal4} RU486(+) PS341 5 μM	11.56	1.50	12.00	0.2609	109.09	20.00	0.00	0.00	0.00		80/80

								Log Rank Value		
**Suppl. Fig. S8g**	**Sample**	**Mean Lifespan (LF) +/- s.e.m. (Days)**		**Median Lifespan +/- s.e.m. (Days)**		**% Median LF vs. control**	**Max (Days)**	**UAS *foxo* ^{H}/UAS *cncC* /Tub^{Gal4} RU486(-)**	**UAS *foxo* ^{H}/UAS *cncC* /Tub^{Gal4} RU486(+)**	**Total Animals Died/Total**
	UAS *foxo* ^{H}/UAS *cncC* /Tub^{Gal4} RU486(-)	42.999	1.905	46.000	1.747	100	76		0.00	112/115
	UAS *foxo* ^{H}/UAS *cncC* /Tub^{Gal4} RU486(+)	9.742	0.230	10.000	0.313	21.73913043	17	0.00		159/159

								Log Rank Value		
**Suppl. Fig. S8h**	**Sample**	**Mean Lifespan (LF) +/- s.e.m. (Days)**		**Median Lifespan +/- s.e.m. (Days)**		**% Median LF vs. control**	**Max (Days)**	**UAS *foxo* ^{H}/ Tub^{Gal4} RU486(+)**	**UAS *foxo* ^{H}/UAS *cncC* /Tub^{Gal4} RU486(+)**	**Total Animals Died/Total**
	UAS *foxo* ^{H}/ Tub^{Gal4} RU486(+)	11.132	0.163	11.000	0.170	100	20		0.00	273/273
	UAS *foxo* ^{H}/UAS *cncC* /Tub^{Gal4} RU486(+)	9.742	0.230	10.000	0.313	90.90909091	17	0.00		159/159
